# CPX-351 vs daunorubicin, cytarabine, and gemtuzumab ozogamicin in older adults with non–adverse-risk AML: the NCRI AML18 trial^∗^

**DOI:** 10.1182/blood.2025031006

**Published:** 2025-11-18

**Authors:** Steven Knapper, Laura W. Dillon, Malavika Babu, Abin Thomas, Ian Thomas, Christopher S. Hourigan, Georgia Andrew, Richard Dillon, Amanda Gilkes, Nuria Marquez Almuina, Sophie King, Nicholas McCarthy, Reem Bahr, Rasha W. Al-Ali, Louisa Stone, Tom Coats, Jennifer Byrne, Simone Green, Ulrik Malthe Overgaard, Rob S. Sellar, Mike Dennis, Priyanka Mehta, Robert Hills, Sylvie D. Freeman, Nigel H. Russell

**Affiliations:** 1 Division of Cancer and Genetics, Cardiff University School of Medicine, Cardiff, United Kingdom; 2 Fralin Biomedical Research Institute, Virginia Tech Fralin Biomedical Research Institute, Cancer Research Center, Washington, DC; 3 Centre for Trials Research, Cardiff University, Cardiff, United Kingdom; 4 Laboratory of Myeloid Malignancies, National Heart, Lung, and Blood Institute, Bethesda, MD; 5 Department of Medical and Molecular Genetics, Kings College London, London, United Kingdom; 6 Department of Clinical Immunology Service, School of Infection, Inflammation and Immunology, University of Birmingham College of Medicine and Health, Birmingham, United Kingdom; 7 Clinical Haematology, Auckland City Hospital, Auckland, New Zealand; 8 Department of Clinical Haematology, Royal Devon and Exeter NHS Foundation Trust, Exeter, United Kingdom; 9 Department of Clinical Haematology, Nottingham University Hospitals NHS Trust, Nottingham, United Kingdom; 10 Department of Clinical Haematology, Castle Hill Hospital, Hull, United Kingdom; 11 Department of Hematology, Copenhagen University Hospital, Rigshospitalet, Copenhagen, Denmark; 12 University College London Cancer Institute, University College London Hospital, London, United Kingdom; 13 Department of Haematology, The Christie NHS Foundation Trust, Manchester, United Kingdom; 14 Department of Haematology, The University of Bristol and Weston NHS Trust, Bristol, United Kingdom; 15 Nuffield Department of Population Health, University of Oxford, Oxford, United Kingdom; 16 Department of Haematology, Guy's and St Thomas' NHS Foundation Trust, London, United Kingdom

## Abstract

We compared daunorubicin/cytarabine plus fractionated gemtuzumab ozogamicin (DAGO2) with CPX-351 (CPX; 1:2 randomization) in 439 patients with acute myeloid leukemia (AML) aged ≥60 years (median age, 68 years) without known adverse-risk cytogenetics. Median follow-up was 35 months. Patients not in measurable residual disease (MRD)-negative remission after course 1 could enter a second randomization between standard and intensified chemotherapy. Post–course 1, the overall response rate (complete remission [CR] + CR with incomplete hematological recovery) was greater after DAGO2 (60% vs 47.5%; odds ratio [OR], 0.61; *P* = .016). Following course 2, the overall response was not significantly different (85% for DAGO2 vs 78% for CPX; *P* = .095). More patients attained CR with MRD negativity after course 1 in the DAGO2 arm (47% vs 29% for CPX; OR, 0.46; *P* = .004). We observed better 3-year event-free survival (34% vs 27%; hazard ratio [HR], 0.73; *P* = .012) and overall survival (52% vs 35%; HR, 0.62; *P* = .001) with DAGO2. CPX did not provide a survival benefit in patients with myelodysplasia (MDS)–related mutations and was associated with poorer survival in patients with *NPM1* (HR, 2.83) and *FLT3* mutations (HR, 2.14). Overall, 37% of patients underwent transplantation in first remission, with no difference in transplantation frequency or survival after transplant between randomization groups. Among patients entering the course 2 randomization (n = 107), survival was equivalent between standard and intensified CPX doses (*P* = .565). In conclusion, in this population of older patients with AML without known adverse-risk cytogenetics, DAGO2 resulted in superior survival compared with CPX. CPX did not benefit patients with MDS-related mutations over DAGO2. This trial was registered at www.ClinicalTrials.gov as #NCT02272478.

## Key Points

• For fit patients with AML aged >60 y without known adverse-risk cytogenetics, we randomized DA chemotherapy plus gemtuzumab against CPX-351.
• Response and survival were better with DAGO, including in those patients with MDS-related mutations.

## Abstract (editor highlights)

4a109f3b-5112-40ca-893e-b436945a467d

## Visual Abstract

**Figure:**
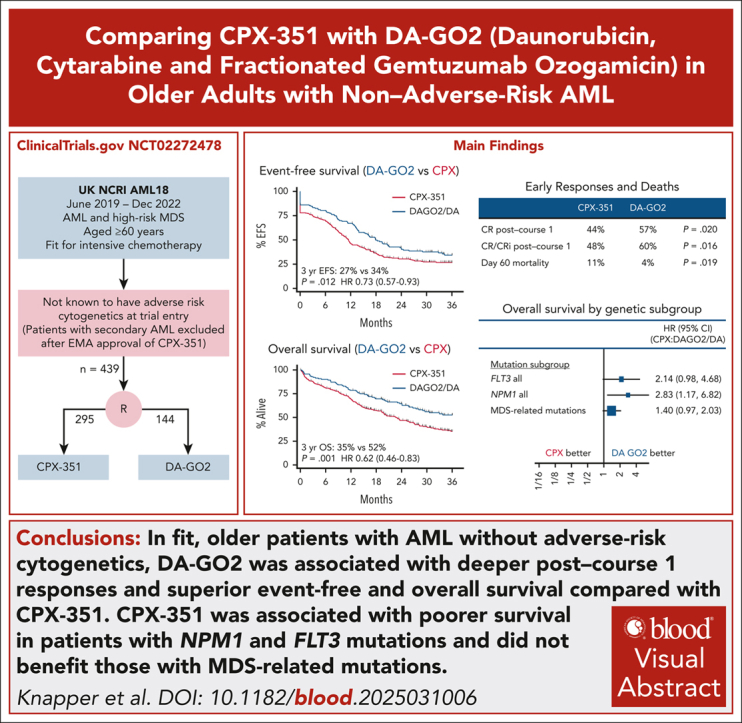

## Teaser

The optimal induction regimen for fit older adults with acute myeloid leukemia (AML) is controversial. Knapper and colleagues report the results of a large, randomized trial of daunorubicin (D) and cytosine arabinoside (A) with 2 doses of gemtuzumab ozogamicin (GO) (DA-GO2) vs liposomal fixed-ratio D and A (CPX-351) in AML without adverse cytogenetics, with DA-GO2 providing superior event-free and overall survival and less prolonged hematological toxicity. Now we need to know how this augmented regimen compares with lower-intensity venetoclax-based regimens.

## Introduction

The UK National Cancer Research Institute (NCRI) Acute Myeloid Leukemia (AML) Working Group has run a sequence of randomized trials aiming to improve the outcome for older adults with AML who are fit for intensive chemotherapy.^1^, ^2^, ^3^ The AML16 trial showed that the addition of a single dose of gemtuzumab ozogamicin (GO) with daunorubicin/cytarabine (AraC; DA) improved overall survival (OS), and this benefit was primarily seen in patients with favorable- or intermediate-risk cytogenetics. In version 1 of the NCRI AML18 trial, we demonstrated that, in older adults, a fractionated schedule of 2 doses of GO (GO2) combined with course 1 DA induction had greater efficacy than a single dose of GO, based on better measurable residual disease (MRD) reduction and superior OS for patients with non–adverse-risk cytogenetics.^2^ This survival benefit was apparent in patients with myelodysplasia (MDS)–related gene mutations as well as in those with de novo AML and was dependent upon delivery of transplant in first remission (CR1). We also demonstrated that, for patients with evidence of residual disease after course 1 of DAGO induction (DA plus GO), survival was improved by course 2 chemotherapy intensification.^4^

In the last few years, evidence has emerged that CPX-351 (CPX), a liposomal formulation of DA improves survival in older patients with newly diagnosed secondary AML (therapy-related AML and AML with MDS-related changes [AML-MRC]) compared with DA chemotherapy.^5^ In version 2 of the AML18 trial (June 2019 to December 2022), we compared DA plus fractionated GO (DAGO2) with CPX for ≤3 courses in patients with AML aged >60 years without known adverse-risk cytogenetics. After the European Medicines Agency approval of CPX for clinically defined secondary AML, these patients were also excluded from trial entry. Because persistence of measurable disease after first induction is associated with an adverse outcome in older patients with AML,^6^ patients who were not in MRD-negative remission by flow cytometry after course 1 of CPX could enter a randomization comparing intensified (3 doses) with standard (2 doses) CPX in course 2.

Here, we report outcomes from this study, which closed after accrual of 439 patients, with recruitment affected by the COVID-19 pandemic. Although the approved use of CPX is for patients with clinical secondary AML or with MDS-related cytogenetic abnormalities, it might also benefit additional patients with AML and MDS-related mutations, as suggested by an exploratory analysis of the AML19 trial comparing of CPX with fludarabine, cytarabine, granulocyte colony-stimulating factor, and idarubicin (FLAG-Ida) in high-risk younger adults.^7^ MDS-related mutations were frequent in version 1 of AML18 trial (∼50% in patients without adverse-risk cytogenetics).^2^ We therefore also evaluated for any differential efficacy between CPX and DAGO2 across relevant molecular subgroups by molecular profiling together with flow cytometric MRD testing.

## Methods

### Patients and trial treatments

The UK NCRI AML18 protocol (ISRCTN-31682779, EudraCR-2013-002730-21) involved several therapeutic questions in previously untreated patients with AML aged ≥60 years without known adverse-risk cytogenetics who were fit for intensive chemotherapy and did not have acute promyelocytic leukemia or blast transformation of chronic myeloid leukemia. Patients with high-risk myelodysplastic syndrome (defined as >10% marrow blasts at diagnosis) were also eligible. Clinical secondary AML was defined as resulting from either an antecedent hematological disorder or prior chemotherapy for a nonhematological malignancy. After the European Medicines Agency approval of CPX for secondary AML, these patients were also excluded from entry into the CPX randomization.

**Figure 1.**
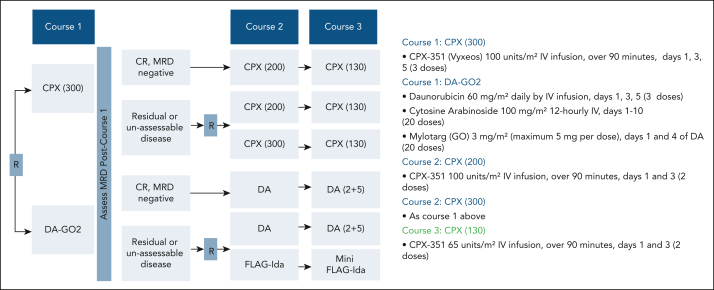
**Trial schema.** CPX total doses (units per square meter) by course are given in brackets (further information in “Methods”). Post–course 1 randomization in DAGO arm for patients with residual or unassessable disease is reported by Russell et al^4^ (further information on FLAG-Ida schedule is provided in “Methods”). CR, complete remission; DA, daunorubicin/AraC; GO2, gemtuzumab ozogamicin, 2 doses; FLAG-Ida, fludarabine, cytarabine, granulocyte colony-stimulating factor, and idarubicin; MRD, measurable residual disease; R, randomization.

Full treatment schedules and trial schema for the CPX randomization are displayed in Figure 1. Patients were assigned on a 1:2 basis to chemotherapy course 1 comprising DA (daunorubicin [60 mg/m^2^ on days 1, 3, and 5] and AraC [100 mg/m^2^ twice daily on days 1-10]) with 2 doses of GO (DAGO2)^2^ or CPX (100 units/m^2^: 100 mg/m^2^ AraC and 44 mg/m^2^ daunorubicin) administered on days 1, 3, and 5 (CPX 300). Patients with abnormal liver function could enter the randomization and receive DA alone if randomized accordingly. In both arms, patients who were not in MRD-negative remission could enter a second-course randomization for treatment intensification. Results of treatment intensification in the DAGO2 arm with FLAG-Ida have been reported.^4^ The daily AraC dose in FLAG-Ida was limited to 1 g/m^2^, and FLAG-Ida was further dose reduced for patients aged >70 years and in course 3 for all patients (fludarabine from 30 mg/m^2^ on days 1-5 to 25 mg/m^2^ days 1-4, idarubicin from 8 mg/m^2^ on days 3-5 to 5 mg/m^2^ on days 2-4). In the CPX arm, the intensification randomization compared a second 3-day course on days 1, 3 and 5 (intensified CPX, CPX 300) with a standard 2-day course on days 1 and 3 (100 units/m^2^; CPX 200; Figure 1). If MRD negative, patients in the CPX arm received 100 units/m^2^ on days 1 and 3 as course 2; those randomized to the DAGO2 arm received DA (3+8) without GO. CPX was reduced to 65 units/m^2^ on days 1 and 3 (CPX 130) for the third course. Patients in the DAGO2 arm who were *FLT3* mutated could also enter a post–course 1 randomization to receive quizartinib (AC220) or not; results of this randomization have been reported elsewhere.^8^ Only 7 patients received quizartinib. Patients were enrolled from 81 centers in the United Kingdom and 6 in Denmark. The study was approved by the ethics committees (all Wales research ethics committee; approved by Danish national and regional ethics bodies for sites in Denmark) and conducted in accordance with Good Clinical Practice guidelines and the Declaration of Helsinki. All patients provided written informed consent for trial entry and for the separate randomizations.

### Laboratory studies

Cytogenetic analyses, performed locally, were reviewed and coded centrally according to the criteria by Grimwade et al.^9^ Mutation analysis of *FLT3* and *NPM1* was performed in a single reference laboratory. Banked diagnostic DNA was analyzed for variants in 75 recurrently mutated myeloid genes (supplemental Methods, available on the *Blood* website). AML with secondary-type mutations (MDS-related mutations) was defined by the presence of ≥1 mutations in *ASXL1*, *BCOR*, *EZH2*, *RUNX1*, *SF3B1*, *SRSF2*, *STAG2*, *U2AF1*, or *ZRSR2*.

MRD was assessed by flow cytometry in a single reference laboratory, as previously described.^2^^,^^4^ Results were entered into the trial database within 24 to 48 hours of sample receipt, blinded to investigator-reported remission status to allow independent refinement of clinical remission assessments. Post–course 1 results were then issued immediately to investigators by the trials unit. Flow MRD testing combined detection of diagnostic leukemic aberrant immunophenotypes (LAIP) and different from normal aberrant immunophenotypes, as per consensus recommendations,^10^^,^^11^ with any measurable level of MRD considered positive (above sensitivity threshold of 0.02%-0.05%). An MRD-negative result required negativity in an adequate bone marrow (BM) sample by both different from normal aberrant immunophenotype and LAIP analysis (prerequisite of LAIP targets identified at baseline). Patients were categorized as MRD unassessable after course 1 if no adequate BM was received before course 2 assignment or in the absence of a baseline LAIP to confirm MRD negativity. Post–course 2 BM samples for MRD were only requested in patients entering the course 2 intensification randomization.

### Statistical considerations and end points

The randomization opened to recruitment in June 2019, with a primary end point of OS, based on a total of 700 patients and ∼440 events. Because of the impact of COVID-19 on recruitment (which reduced from ∼30 patients per month in 2019 to <10 patients per month throughout the pandemic), the primary end point was amended in December 2021 to event-free survival (EFS), incorporating relapse and resistance to treatment as well as death into the definition of an event,^12^^,^^13^ with a target of 506 patients. The randomization closed in December 2022, under authorization from the independent data monitoring committee, having accrued 439 patients.

Analyses were by intention to treat. End points were defined according to the revised International Working Group criteria.^14^ Responses were based on investigator assessment of BM samples. Complete remission (CR) and CR with incomplete hematological recovery (CRi; ≤50 days for course 1 response and ≤100 days for induction response) were combined for outcome analyses. Characteristics of the patients are summarized across the group using frequency and percentage for categorical data, and median and quartile range for quantitative data. Comparisons of patient characteristics were made using χ^2^ tests, Mantel-Haenszel tests for trend, or Wilcoxon rank-sum tests, as appropriate. Time-to-event outcomes were compared using log-rank tests and Cox regression. Outcomes were reported as effect sizes with 95% confidence intervals (CI); significance was set at *P* value <.05. EFS was measured in all patients and was defined as time from randomization to treatment failure (refractory disease or partial response by the end of course 2), relapse, or death from any cause. If treatment failure was because of refractory disease or partial response, the event was recorded on cycle 1 day 1. OS was defined as the time from randomization to death from any cause, with those still alive censored at the date last seen. Relapse-free survival (RFS) was calculated only for patients who achieved CR or CRi, and was measured from the date of CR/CRi until the date of relapse or death from any cause. Competing-risk analysis was performed for the cumulative incidence of relapse and cumulative incidence of death in remission, with nonrelapse mortality and hematological relapse, respectively, as the competing risk, using the Gray test and Fine and Gray model. For the exploratory analyses of key subgroups, hazard ratios (HR) were calculated using Cox proportional hazards models and represented in forest plots, with a test for trend of heterogeneity across the subgroups, wherever applicable.

Toxicity (hematological recovery times and nonhematological toxicity) was scored using the National Cancer Institute Common Toxicity Criteria, version 3.^15^ Resource-use data (blood product support, days on antibiotics, and hospitalization) were collected.

## Results

### Patient characteristics

**Figure 2.**
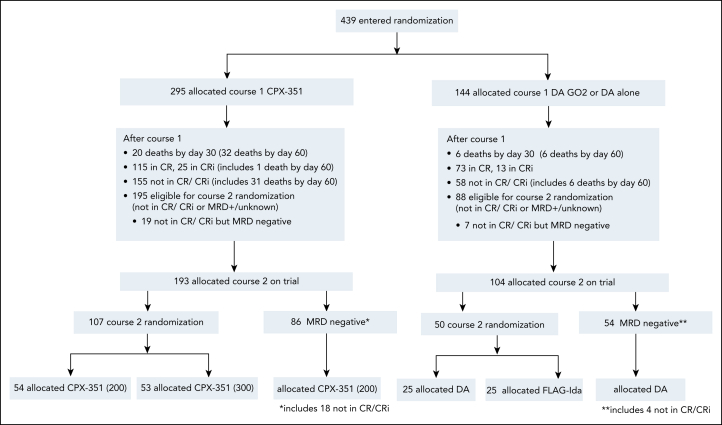
**CONSORT diagram.**

From June 2019 to December 2022, 439 patients were randomized 2:1 in favor of CPX (CPX, n = 295; DAGO2, n = 144, including 7 with DA alone; CONSORT, Figure 2). Clinical baseline characteristics of the patients are shown in Table 1 and were generally balanced between the treatment arms. There was no significant difference between arms for European LeukemiaNet (ELN) genetic risk or mutation subgroups (*P* value not significant for all). Failed or unknown cytogenetic results were more frequent in the DAGO2 arm (*P* = .029). The median age was 68 years, with 33% of patients aged >70 years; 81% had clinical de novo AML, 7% clinical secondary AML (entered before the amendment that excluded these from randomization), and 12% had high-grade MDS. Because some patients in this study were randomized before their cytogenetic results were known, the cohort eventually included 8% with adverse cytogenetics. In total, 404 patients had mutation panel data (supplemental Figure 1), *FLT3* mutations were present in 16%, *NPM1* mutations in 24%, *TP53* mutations in 4%, and MDS-related gene mutations (secondary AML mutations) were present in 60%. *DDX41* mutations were present in 9%. Median follow-up was 35 months.

**Table 1.** **Patient demographics and clinical characteristics**

	Overall N = 439	CPX n = 295	DAGO2 or DA n = 144
**Age, median (range), y**	68 (64-71)	68 (64-70)	67 (64-71)
≥65	310 (70.6)	209 (70.9)	101 (70.1)
≥70	147 (33.5)	97 (32.9)	50 (34.7)
Male	262 (59.7)	174 (59.0)	88 (61.1)
**WBC, median (range), ×10^9^/L**	3.5 (1.7-13.6)	3.3 (1.7-13.2)	4.4 (1.6-14.6)
<10	304 (69.3)	205 (69.5)	99 (68.8)
≥50	31 (7.1)	21 (7.1)	10 (6.9)
**Diagnosis**			
Clinical de novo AML	357 (81.3)	240 (81.4)	117 (81.3)
Clinical secondary AML	30 (6.8)	20 (6.8)	10 (6.9)
High-risk MDS	52 (11.9)	35 (11.8)	17 (11.8)
**Performance status (ECOG)**			
0	211 (48.1)	143 (48.5)	68 (47.2)
1	198 (45.1)	132 (44.7)	66 (45.8)
2	30 (6.8)	20 (6.8)	10 (6.9)
**Small molecule from course 2**			
Long quizartinib	4 (2.4)	0 (0.0)	4 (6.9)
Short quizartinib	3 (5.2)	0 (0.0)	3 (5.2)
No quizartinib	158 (95.8)	107 (100.0)	51 (87.9)
**Genetic risk**			
Cytogenetic (Grimwade et al^9^)			
Favorable	4 (1.0)	2 (0.7)	2 (1.4)
Intermediate	346 (81.8)	242 (85.5)	104 (74.3)
Adverse	32 (7.6)	20 (7.1)	12 (8.6)
Failed	20 (4.7)	8 (2.8)	12 (8.6)
Not reported	21 (5.0)	11 (3.9)	10 (7.1)
ELN 2022			
Favorable	97 (27.2)	63 (25.8)	34 (30.1)
Intermediate	71 (19.9)	48 (19.7)	23 (20.4)
Adverse	189 (52.9)	133 (54.5)	56 (49.6)
Unknown/missing	82	51	31
**Mutations**	n = 404	n = 273	n = 131
*FLT3*-ITD/TKD	66 (16.3)	45 (16.5)	21 (16.0)
*NPM1*	97 (24.0)	66 (24.2)	31 (23.7)
MDS-related	243 (60.1)	165 (60.4)	78 (59.5)
MDS-related, excluding *FLT3/NPM1*	199 (49.3)	136 (49.8)	63 (48.1)
*TP53*	17 (4.2)	14 (5.1)	3 (2.3)
Unknown	35	22	13

Data are presented as number (%) unless otherwise specified.

ECOG, Eastern Cooperative Oncology Group; ELN, European LeukemiaNet; TKD, tyrosine kinase domain; WBC, white blood cells.

### Response and outcome

Following course 1, overall (CR + CRi) and CR rates were higher after DAGO2 (CR + CRi, 60% vs 47.5% [odds ratio (OR), 0.61; 95% CI, 0.41-0.91; *P* = .016]; CR, 50.5% vs 39% [OR, 0.62; 95% CI, 0.42-0.93; *P* = .020]). Following course 2, the overall response was not significantly different (85% for DAGO2 vs 78% for CPX; OR, 0.64; 95% CI, 0.39-1.09; *P* = .095), although there was a higher frequency of CR with DAGO2 (78.5% vs 68%; OR, 0.59; 95% CI, 0.37-0.94; *P* = .024; Table 2). Next, we assessed the impact on MRD reduction measured by flow cytometry. Significantly lower MRD levels were observed in DAGO2 patients compared to CPX (supplemental Figure 2A) in all patients evaluable for response by flow cytometric MRD (supplemental Figure 2B). Of the randomized patients, 404 were assessable for composite response that included MRD for those in CR and CRi after first induction (Table 2). Among these, CR without MRD based on ELN criteria (MRD, <0.1%) was achieved by 47% and 29% of patients assigned to DAGO2 and CPX, respectively (*P* = .004; Table 2). MRD response data were available in 84.5% of patients in CR/CRi after course 1 (CPX, 114/140; DAGO, 73/86; Table 2). Of these, more attained MRD <0.1% in the DAGO2 arm (85% vs 68% for CPX; OR, 0.37; 95% CI, 0.16-0.82; *P* = .008; Table 2). Distribution of flow cytometric MRD responses by treatment arm in specific mutation subgroups are displayed in supplemental Figure 2C. Increased efficacy in MRD was observed for DAGO2 compared with CPX across mutation groups, including MDS-related mutations.

**Table 2.** **Response, early deaths, and rates of allogeneic stem cell transplantation**

	CPX	DAGO2 or DA	*P* value	OR (95% CI)
**Response**	n = 295	n = 144		
CR + CRi	230 (78.0)	122 (84.7)	.095	0.64 (0.37-1.09)
CR	201 (68.1)	113 (78.5)	.024	0.59 (0.37-0.94)
CR + CRi after course 1	140 (47.5)	86 (59.7)	.016	0.61 (0.41-0.91)
CR after course 1	115 (39)	73 (50.7)	.020	0.62 (0.42-0.93)
**Response including flow cytometric MRD status after course 1**	n = 273∗	n = 131∗		
CR/CRi MRD <0.1%	80 (29.3)	62 (47.3)	.004	0.46 (0.29-0.73)
CR MRD <0.1%	78 (28.6)	61 (46.6)	.004	0.46 (0.29-0.72)
CR/CRi MRD negative	68 (24.9)	50 (38.2)	.006	0.54 (0.34-0.86)
CR MRD negative	66 (24.2)	48 (36.6)	.009	0.66 (0.34-0.89)
**Post–course 1 CR/CRi with MRD result**	n = 118†	n = 73†		
MRD <0.1%	80 (67.8)	62 (84.9)	.008	0.37 (0.16-0.82)
CR MRD <0.1%	78 (66.1)	61 (83.6)	.008	0.38 (0.17-0.83)
MRD negative	68 (57.6)	50 (68.5)	.134	0.63 (0.34-1.16)
CR MRD negative	66 (55.9)	48 (65.8)	.180	0.66 (0.36-1.21)
**Early death**				
Day 30	20 (6.8%)	6 (4.2%)	.276	1.67 (0.66-4.26)
Day 60	32 (10.9)	6 (4.2)	.019	2.80 (1.14-6.86)
**Allogeneic Stem Cell Transplant**	108 (36.6)	54 (37.5)	.856	0.96 (0.64-1.45)
Allograft in CR1	93 (40.4)	44 (36.1)	.424	1.20 (0.76-1.89)
Time to allograft in CR1, median (range), d‡	127 (84-190)	137 (80-190)	.478	

MRD was measured by flow cytometry and categorized as MRD negative (<0.1%) or detectable but <0.1%. *P* values were calculated using χ^2^ or Fisher exact test.

∗ All evaluable patients include those in CR or CRi with MRD data, as well as all patients who did not attain CR/CRi, including day 30 deaths.

† Evaluable patients are those in CR or CRi with available MRD data.

‡ Patients receiving CR1 transplant.

**Figure 3.**
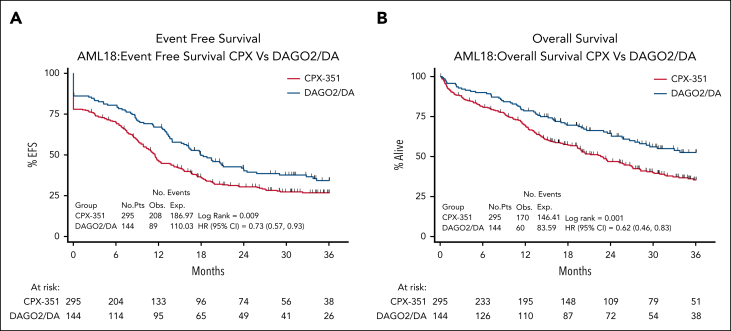
**Survival outcomes by randomization.** (A) EFS. (B) OS.

Both EFS (HR, 0.73; 95% CI, 0.57-0.93; *P* = .012) and OS (HR, 0.62; 95% CI, 0.46-0.83; *P* = .001) were better with DAGO2. With a median follow-up of 35 months, EFS and OS at 3 years were 34% and 52% for DAGO2 compared with 27% and 35% for CPX (Figure 3). RFS was also higher with DAGO2, although this did not reach significance (HR, 0.75; 95% CI, 0.57-1.01; *P* = .058). There was no significant difference between treatment arms in survival from remission (HR, 0.74; 95% CI, 0.48-1.13; *P* = .162), cumulative incidence of relapse (subdistribution hazard ratio, 0.87; 95% CI, 0.56-1.31; *P* = .52), or cumulative incidence of death in CR (subdistribution hazard ratio, 1.05; 95% CI, 0.43-2.54; *P* = .92) for those patients who attained CR/CRi by day 50 (supplemental Figure 3).

A sensitivity analysis that excluded patients with adverse cytogenetics or TP53-mutated AML confirmed better survival with DAGO2 in patients with favorable and intermediate-risk cytogenetics (supplemental Figure 4).

**Figure 4.**
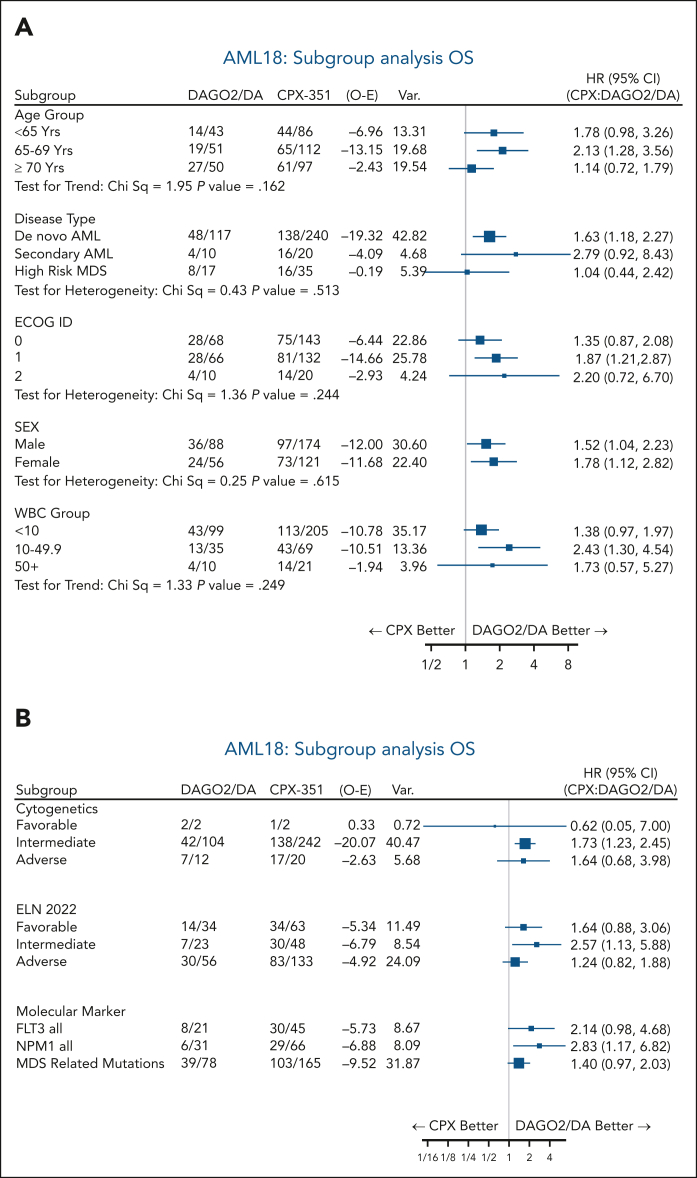
**Subgroup analysis of OS.** (A) Patient characteristics. (B) Baseline genetics. MDS-related mutation subgroup includes patients with NPM1 or FLT3-ITD/TKD mutations. ECOG, Eastern Cooperative Oncology Group; ELN, European LeukemiaNet; O-E, observed to expected; Var., variance; WBC, white blood cells.

**Figure 5.**
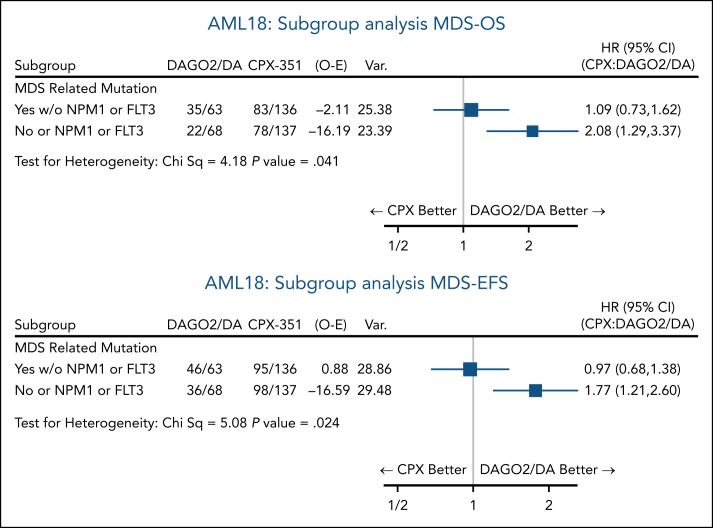
**Forest plot of OS by randomization for patient subgroup with MDS-related mutations, excluding NPM1 or FLT3-ITD/TKD mutations, vs others.** O-E, observed to expected; Var., variance.

The results for EFS and OS at 3 years from the time of randomization favored DAGO2 in all subgroups based on clinically defined baseline characteristics (age, white blood cell count, disease type, and cytogenetics; Figure 4; supplemental Figure 5A). In a stratified analysis, CPX did not provide an OS benefit over DAGO2 in patients with MDS-related mutations (HR, 1.40; 95% CI, 0.97-2.03) and was associated with worse OS in patients with *NPM1* (HR, 2.83; 95% CI, 1.17-6.82) and *FLT3* mutations (HR, 2.14; 95% CI, 0.98-4.68; Figure 4B; EFS results shown in supplemental Figure 5B). The 3-year OS in patients with *NPM1* mutations was 78% with DAGO2 compared with 51% with CPX. Similar results were observed when the 7 patients in the DAGO2 arm receiving quizartinib from course 2 were excluded from the analysis (supplemental Figure 6). In exploratory subgroup analysis, there was less OS benefit from DAGO2 compared to CPX for patients with MDS-related mutations and wild-type *NPM1*/*FLT3* (HR, 1.09; 95% CI, 0.73-1.62) vs other patients (HR, 2.08; 95% CI, 1.29-3.37; *P* value for heterogeneity 0.038; Figure 5).

The 3-year OSs was 49% for patients with *DDX41* mutations, with no detectable difference by randomization.

In total, 162 (37%) patients received an allogeneic stem cell transplant; most transplants (n = 137 [85%]) were undertaken in CR1 (transplants, 44/142 [36%] in the DAGO2 arm and 93/230 [40%] in the CPX arm; Table 2). Survival after CR1 transplant did not differ by randomization (OS: HR, 0.98; 95% CI, 0.52-1.85). OS and RFS at 3 years after transplant were 57% and 55%, respectively, for DAGO2, compared with 64% and 61%, respectively, for CPX (supplemental Figure 7).

### Treatment toxicity and resource usage

Hematological recovery was slower with CPX than with DAGO2 (supplemental Table 1). After course 1, the time to neutrophil recovery to 1 × 10^9^/L was 31 and 34 days for DAGO2 and CPX, respectively (*P* = .033). Likewise, platelet count was delayed with CPX, the time to platelet recovery to 100 × 10^9^/L was 31 and 34 days for DAGO2 and CPX, respectively (*P* = .02). With respect to supportive care, days of antibiotic use, or hospitalization, and the time to the start of course 2 did not differ between the CPX and DAGO2 arms. Gastrointestinal toxicity was greater with DAGO2 (supplemental Figure 8). Early all-cause mortality was not different at day 30 between DAGO2 and CPX (4% and 7%; HR, 1.67; 95% CI, 0.66-4.26; *P* = .27) but was higher with CPX at day 60 (4% vs 11%; HR, 2.80; 95% CI, 1.14-6.86; *P* = .019). The increased early mortality was mainly related to infection and disease (supplemental Figure 8).

### Outcomes of high-risk randomization in CPX arm

After course 1, a total of 157 patients entered the high-risk randomizations for patients not in an MRD-negative remission (107 after CPX induction and 50 after DAGO2 induction; Figure 2, CONSORT). Baseline characteristics of the patients are shown in supplemental Table 2 and were balanced between the treatment arms. Outcomes for patients randomized to DAGO2 have previously been reported.^4^ There was no evidence that intensified CPX increased rates of conversion to CR from refractory disease or increased MRD negativity for patients in an MRD-positive CR after course 1 (supplemental Table 3). Likewise, there was no evidence of a survival benefit (supplemental Figure 9).

Of all patients who were eligible for the high-risk course 2 randomization (excluding early deaths), 126 did not enter (CPX, 88/195 eligible; DAGO2, 38/88 eligible), including 102 patients not in remission after course 1. Of these, 43% (33/76) attained CR/CRi in the CPX arm and 27% (7/26) in the DAGO2 arm within 100 days.

## Discussion

Previous studies with CPX have primarily focused on patients with high-risk AML, in whom a survival benefit has been shown when compared with standard DA induction in patients with clinically defined secondary disease or MDS-related cytogenetic abnormalities.^5^ This survival benefit was ascribed to a favorable toxicity profile, higher rates of transplantation, and superior posttransplant survival.^5^ We considered that these benefits might also extend to older patients without these adverse-risk factors; however, based on our recent encouraging experience in the NCRI AML18 version 1 trial, we compared CPX with DAGO2 rather than with DA alone.^2^ The randomization recruitment target was not reached because of the impact of the COVID-19 pandemic (supplemental Figure 10). Nonetheless, the results of this comparison show that in fit older patients, primarily with clinical de novo AML and intermediate-risk cytogenetics, CPX did not improve survival outcomes compared with DAGO2, overall or in any subgroup, including those with MDS-related mutations. DAGO2 induction resulted in higher rates of hematological and flow MRD response from first induction and lower 60-day mortality. There was less hematological toxicity with DAGO2, although there was greater grade 1 to 2 gastrointestinal toxicity. The risks of relapse and death in remission were similar between the 2 treatment arms, and the improvement in OS and EFS was primarily because of the superior and deeper early response and better tolerability. The advantage for DAGO2 was particularly apparent in patients with de novo AML-type mutations, specifically *NPM1* and *FLT3.* There was only a trend toward a survival benefit with DAGO2 in the MDS-related mutation group, which was not present when patients with comutations in *NPM1* and *FLT3* were excluded. The more advantageous DAGO2 response observed in the MDS-related mutation group with comutated *NPM1*, as opposed to MDS-related mutations without *NPM1,* is consistent with other studies indicating that the *NPM1* mutation is the primary determinant of outcome in patients with mutations in both *NPM1* and MDS-related genes, and therefore should guide induction decisions.^16^, ^17^, ^18^, ^19^ In the NCRI AML19 trial, we previously observed a superior outcome with CPX compared with FLAG-Ida in high-risk patients with MDS-related mutations.^7^ This benefit was primarily because of less hematological toxicity and lower mortality after the second course of CPX compared with FLAG-Ida, with more patients received transplants after CPX and superior posttransplant outcomes. In AML18, we did not observe a benefit for CPX in patients with MDS-related mutations even when patients with coexisting *NPM1* and *FLT3* mutations were excluded from the analysis, suggesting that DAGO2 was better tolerated in this older adult population than was FLAG-Ida in our younger adult AML19 trial. Furthermore, in this study, transplant rates in CR1 with DAGO2 and CPX were comparable, with no difference in posttransplant survival by randomization.

Interestingly, although remission rates after course 1 were significantly lower in the CPX arm, overall remission rates were not. Furthermore, the inferior MRD response after course 1 in CPX patients did not appear to reduce posttransplant OS and RFS compared to DAGO2. This differs from the previously observed benefit of early efficacy on posttransplant outcomes from DAGO2 compared to GO1 in AML18 version 1.^2^ A limitation of this study is the unavailability of post–course 2 MRD results and treatment information for patients who did not enter the high-risk course 2 randomizations. Therefore, we could not assess whether CPX is associated with slower response kinetics or if off-trial chemotherapy compensated for the poorer cytoreduction following course 1 CPX in patients who proceeded to transplant. However, course 2 intensification with 3 vs 2 doses of CPX did not improve survival in patients without an MRD-negative remission after course 1. This contrasts with our experience of the benefit of postinduction treatment intensification with FLAG-Ida or cladribine with DA after initial DAGO2.^4^

Notably, the population studied here, consisting primarily of older fit patients with clinical de novo AML, was very different from that in the pivotal trial with secondary AML, in which a survival benefit of CPX compared with DA was reported.^5^ Our patients were older and excluded those with known adverse-risk cytogenetics or a history of antecedent hematological disease, focusing primarily on patients with more chemotherapy-sensitive disease, in whom outcomes with DAGO2 are very encouraging, with survival exceeding 50% at 3 years. In this cohort, compared with reported results in patients with high-risk/secondary AML receiving CPX, overall response rates with CPX were improved.^5^^,^^20^ Indeed, the survival of patients receiving CPX here was comparable to that seen with DA and a single dose of GO in the first version of AML18.^2^ The improved survival of patients receiving DAGO2 compared to that seen in AML18 version 1 may reflect both reduced early mortality and the increased number of patients received transplants in first remission over the course of the trial, reaching 36% for DAGO2 compared with 25% in AML18 version 1. We note that FLT3 inhibitors were not available as standard of care during the study, and the combination with CPX is currently under investigation^21^ (ClinicalTrials.gov identifier: NCT04293562). Further possible limitations to this study include the possible impact of the COVID-19 pandemic on outcomes (although no early deaths were attributed to COVID-19) and GO could not be included in the CPX arm, precluding direct comparison of CPX GO with DAGO2. Potentially, the addition of GO to CPX might achieve higher early response rates to those reported here; this has been explored,^22^^,^^23^ including a recent randomized pediatric AML study in which CPX GO (comparison with DAGO) was associated with inferior 2-year EFS and relapse incidence along with increased risk of hematological toxicity.^23^

In summary, in this study of older patients, primarily with clinical de novo AML and intermediate-risk cytogenetics, DAGO2 resulted in superior survival compared to CPX, including in patients with MDS-related mutations. This was consistent with the observed difference in MRD measured early leukemia clearance, thus supporting MRD as an early clinical end point in AML.

Conflict-of-interest disclosure: N.H.R. reports honoraria from Jazz Pharmaceuticals, Pfizer, and Astellas Pharma; research funding from Jazz Pharmaceuticals (to the institution); and travel, accommodations, and expenses from Jazz Pharmaceuticals. R.D. reports research support from AbbVie, Amgen, Jazz Pharmaceuticals, and Pfizer; travel support from Servier and Jazz Pharmaceuticals; consulting roles with AbbVie, Astellas, Jazz Pharmaceuticals, Pfizer, and Servier; and membership on the data safety and monitoring board for AvenCell. C.S.H. reports research support from Illumina and a consulting role with Astellas Pharma. P.M. reports honoraria from Astellas Pharma, Pfizer, Jazz Pharmaceuticals, AbbVie, and Servier; consulting or advisory roles with Jazz Pharmaceuticals and AbbVie; membership in the speakers' bureau of Jazz Pharmaceuticals, AbbVie, and Astellas Pharma; and travel, accommodations, and expenses from Jazz Pharmaceuticals. T.C. reports speaker/advisory board fees from Astellas Pharma, Janssen, and Jazz Pharmaceuticals; travel sponsorship from Jazz Pharmaceuticals; and an independent educational grant from Pfizer. J.B. reports advisory roles with Jazz Pharmaceuticals and research funding from Celgene (to the institution), Daiichi Sankyo (to the institution), and Bio-Cancer Treatment International. S.D.F. reports consulting or advisory roles with the MRD Partnership and Alliance in AML Clinical Treatment; membership in the speakers' bureau of Novartis and Jazz Pharmaceuticals; and research funding from Jazz Pharmaceuticals (to the institution), Bristol Myers Squibb/Celgene (to the institution), AstraZeneca (to the institution), and Cytek (to the institution). S. Knapper reports research support from Novartis; travel support from Servier; and consulting fees from AbbVie, Astellas, Jazz Pharmaceuticals, Novartis, Pfizer, and Servier. L.W.D. reports honoraria from Bio-Rad Laboratories. The remaining authors declare no competing financial interests.
