# Supplementary material for: CPX-351 vs daunorubicin, cytarabine, and gemtuzumab ozogamicin in older adults with non–adverse-risk AML: the NCRI AML18 trial
Source: Blood. 2025 Nov 18;147(10):1048–57. doi: 10.1182/blood.2025031006 (PMC13052346; doi:10.1182/blood.2025031006)
Supplement: Supplemental Tables, Figures, and Methods [file BLOOD_BLD-2025-031006-mmc1.pdf]

CPX-351 versus daunorubicin, cytarabine plus gemtuzumab ozogamicin in older adults with non-adverse risk AML: NCRI AML18 trial

Supplementary Material

Table of Contents

SUPPLEMENTARY TABLES 3

TABLE S1 RECOVERY TIMES AND RESOURCE USAGE BY TREATMENT ARM 3
TABLE S2 PATIENTS ENTERING CPX COURSE 2 RANDOMIZATION: DEMOGRAPHICS AND CLINICAL CHARACTERISTICS 5
TABLE S3. PATIENTS ENTERING CPX COURSE 2 RANDOMIZATION: RESPONSE, RATES OF ALLOGENEIC STEM CELL TRANSPLANTATION 7

SUPPLEMENTARY FIGURE LEGENDS 8

FIGURE S1. HEATMAP OF MUTATION VARIANTS PER PATIENT 8
FIGURE S2. FLOW CYTOMETRIC MRD POST COURSE 1 8
A. FLOW CYTOMETRIC MRD LEVELS IN BM POST COURSE 1 ACCORDING TO TREATMENT ARM. 8
B. DISPOSITION OF MRD DATA POST COURSE 1 AND FOR PATIENTS ENTERING COURSE 2 INTENSIFICATION RANDOMISATION 8
C. FLOW CYTOMETRIC MRD RESPONSE RATES POST COURSE 1 BY MOLECULAR SUBTYPES ACCORDING TO TREATMENT ARM. 8
FIGURE S3. OUTCOMES IN PATIENTS ACHIEVING REMISSION BY DAY 50 8
A. OVERALL SURVIVAL FROM REMISSION B. CUMULATIVE INCIDENCE OF RELAPSE 8
C. CUMULATIVE INCIDENCE OF DEATH IN REMISSION 8
FIGURE S4. EVENT-FREE AND OVERALL SURVIVAL OF PATIENTS WITHOUT ADVERSE CYTOGENETICS OR MUTATED TP53. 8
A. EVENT-FREE SURVIVAL OF PATIENTS WITHOUT ADVERSE CYTOGENETICS OR MUTATED *TP53.* 8
B. OVERALL SURVIVAL OF PATIENTS WITHOUT ADVERSE CYTOGENETICS OR MUTATED *TP53* 8
FIGURE S5. SUBGROUP ANALYSIS OF EVENT-FREE SURVIVAL 8
A PATIENT CHARACTERISTIC. B. BASELINE GENETICS 8
FIGURE S6 GENETIC SUBGROUP ANALYSIS EXCLUDING PATIENTS RECEIVING QUIZARTINIB 9
A. OVERALL SURVIVAL. B. EVENT-FREE SURVIVAL 9
FIGURE S7. OVERALL SURVIVAL BY TREATMENT ARM OF PATIENTS WHO RECEIVED AN ALLOGENEIC STEM CELL TRANSPLANTATION IN CR1 9
FIGURE S8. ADVERSE EVENTS (INTENTION TO TREAT POPULATION). 9

Figure S9. Overall survival in patients in CPX treatment arm entering course 2 randomization **9**
A. All patients B. Excluding patients with unknown MRD 9
Figure S10. Patient accrual per month **9**

FIGURES **10**

SUPPLEMENTARY METHODS **23**

AML18 LIST OF SITES AND INVESTIGATORS **27**

Supplementary Tables

Table S1 Recovery Times and Resource Usage by Treatment Arm

	CPX	DAGO2 or DA	P value*
Neutrophil recovery Time from start of Course 1<br>Days, median [IQR]			
All patients	34 [29, 39]	31 [27, 36]	0.033
Clinical Secondary AML	32 [29, 37]	32.5 [28.5, 42.5]	0.653
With MDS related mutations (All)	34 [29, 39]	31 [27, 36]	0.072
With MDS related mutations (*FLT3/NPM1*wt only)	35 [30.5, 41.5]	32 [27.5, 38.5]	0.092
Platelet recovery Time from start of Course 1<br>Days, median [IQR]			
All patients	34 [28, 41]	31 [28, 34]	0.020
Clinical Secondary AML	30 [27, 42]	31 [27, 38]	0.628
With MDS related mutations (All)	34 [29, 40]	31 [27, 35]	0.113
With MDS related mutations (*FLT3/NPM1*wt only)	35 [30, 42]	31 [29, 40]	0.155
Resource usage			
Units of blood	10 [7, 13]	8 [5, 12]	0.002
Units of platelets	9 [6, 13]	11 [7, 16]	0.010
Days of IV antibiotics	21 [14, 27]	19 [15, 26]	0.333
Days of oral antibiotics	6 [0, 15]	7 [0, 17]	0.875
Nights in hospital	36 [29, 44]	35 [31, 43]	0.941

Time to Course 2<br>Days, median [IQR]*	55 [47, 62]	51 [47, 57]	0.078

Table S2 Patients entering CPX course 2 randomization: Demographics and Clinical Characteristics

	Overall	CPX-200	CPX-300
	N=107	N=54	N=53
**Age** median (range)			
Age ≥ 65yrs	76 (71.0)	37 (68.5)	39 (73.6)
Age ≥ 70yrs	36 (33.6)	17 (31.5)	19 (35.9)
**Male**	64 (59.8)	32 (59.3)	32 (60.4)
**WBC** x 10^9 / L median (range)			
<10	84 (95.5)	41 (93.2)	43 (97.7)
≥ 50	4 (4.5)	3 (6.8)	1 (2.3)
**Diagnosis**			
Clinical De Novo AML	89 (83.2)	44 (81.5)	45 (84.9)
Clinical Secondary AML	7 (6.5)	4 (7.4)	3 (5.7)
High Risk MDS	11 (10.3)	6 (11.1)	5 (9.4)
**Performance ID** (ECOG)			
0	63 (58.9)	31 (57.4)	32 (60.4)
1	40 (37.4)	20 (37.0)	20 (37.7)
2	4 (3.7)	3 (5.6)	1 (1.9)
**Small Molecule from Course 2**			
Long quizartinib	0	0	0
Short quizartinib	0	0	0
No quizartinib	107 (100.0)	54 (100.0)	53 (100.0)
**Genetic risk**			
**Cytogenetic** (Grimwade2010)			
Favourable	1 (0.9)	0	1 (1.9)
Intermediate	90 (84.9)	46 (86.8)	44 (83.0)
Adverse	7 (6.6)	3 (5.7)	4 (7.6)

Failed	3 (2.8)	2 (3.8)	1 (1.9)
Not reported	5 (4.7)	2 (3.8)	3 (5.7)
**ELN 2022**			
Favourable	19 (22.1)	10 (23.3)	9 (20.9)
Intermediate	17 (19.8)	13 (30.2)	4 (9.30)
Adverse	50 (58.1)	20 (46.5)	30 (69.8)
Unknown	21	11	10
**Mutations**	**N=96**	**N=47**	**N=49**
FLT3 mutations	17 (17.7)	9 (19.2)	8 (16.3)
NPM1 mutations	14 (14.6)	10 (21.3)	4 (8.2)
MDS-related mutations	62 (64.6)	27 (57.5)	35 (71.4)
MDS-related mutations excluding *FLT3/NPM1*	52 (54.2)	22 (46.8)	30 (61.2)
TP53+	3 (3.1)	0 (0.0)	3 (6.1)
Unknown	11	7	4

Table S3. Patients entering CPX course 2 randomization: Response, Rates of Allogeneic Stem Cell Transplantation

	CPX 200	CPX 300	
**Response conversion after course 2** by day 50 from randomization			P value
Not in CR/CRi post course 1	n=21	n=27	
ORR (CR+CRi) post course 2	16 (76.2%)	17 (63%)	0.327
CR post course 2	15 (71.4%)	17 (63%)	0.537
CRi post course 1(denominator)	n=3	n=1	
CR post course 2	0 (0.0%)	0(0.0%)	-
*MRD conversion**	n=27	n=26	
MRD negative post course 2**	12 (44.4%)	11 (42.3%)	0.875
**Allografts in CR1**	16 (32.0%)	23 (46.0%)	0.151

*patients with MRD results post course 1 and 2

**includes 4 patients (3 CPX 300, 1 CPX 200) not in CR/CRi post course 2

Legend. CR, complete remission; CRi , CR with incomplete count recovery; ORR, overall response rate; CR1, first remission.

Supplementary Figure Legends

Figure S1. Heatmap of mutation variants per patient

Figure S2. Flow Cytometric MRD post course 1

***A.* Flow cytometric MRD levels in BM post course 1 according to treatment arm.**

Median levels, presented as a percentage, were significantly lower in CPX arm (median, 25-75% quartiles with 1-99 percentiles are shown). Comparisons were performed using the Mann-Whitney U test for continuous variables. Results represent all patients with MRD data (including those not in CR/CRi post course 1).

***B.* Disposition of MRD data post course 1 and for patients entering course 2 intensification randomisation**

C. Flow cytometric MRD response rates post course 1 by Molecular Subtypes according to treatment arm.

Denominator is all patients with MRD data (including patients not attaining CR/CRi post course 1).
MRD-ve, CR/CRi post course 1 with MRD not detectable; MRD low +ve, CR/CRi post course 1 with MRD detected but <0.1%; MRD+ve, CR/CRi post course 1 with MRD≥0.1% (ELN MFC-MRD threshold).

***Figure S3.* Outcomes in patients achieving remission by day 50**

A. Overall Survival from remission B. Cumulative Incidence of Relapse

C. Cumulative Incidence of death in remission

Figure S4. Event-free and overall survival of patients without adverse cytogenetics or mutated TP53*.*

A. Event-free Survival of patients without adverse cytogenetics or mutated *TP53.*

B. Overall Survival of patients without adverse cytogenetics or mutated *TP53*

***Figure S5.* Subgroup Analysis of Event-free Survival**

A Patient characteristic. B. Baseline genetics

MDS-related mutation subgroup includes patients with *NPM1* or *FLT3* ITD/TKD mutations

***Figure S6* Genetic Subgroup Analysis excluding patients receiving quizartinib**

A. Overall Survival. B. Event-free Survival

Figure S7.   Overall survival by treatment arm of patients who received an allogeneic stem cell transplantation in CR1, landmarked from the date of transplantation.

Figure S8. Adverse Events (intention to treat population).

The percentage of patients with grades 1-5 events are shown for adverse events by treatment arm. Inset table shows adverse event early deaths (day 60 deaths) by treatment arm

Figure S9.   Overall survival in patients in CPX treatment arm entering course 2 randomization (for patients not achieving a post course 1 MRD negative remission) by course 2 randomization.

A.   All patients   B. Excluding patients with unknown MRD

Figure S10. Patient accrual per month

showing significant drop in accrual from Q2 of 2020 due to COVID pandemic

Figures

Supplementary Figure S1

Heatmap of mutation variants per patient

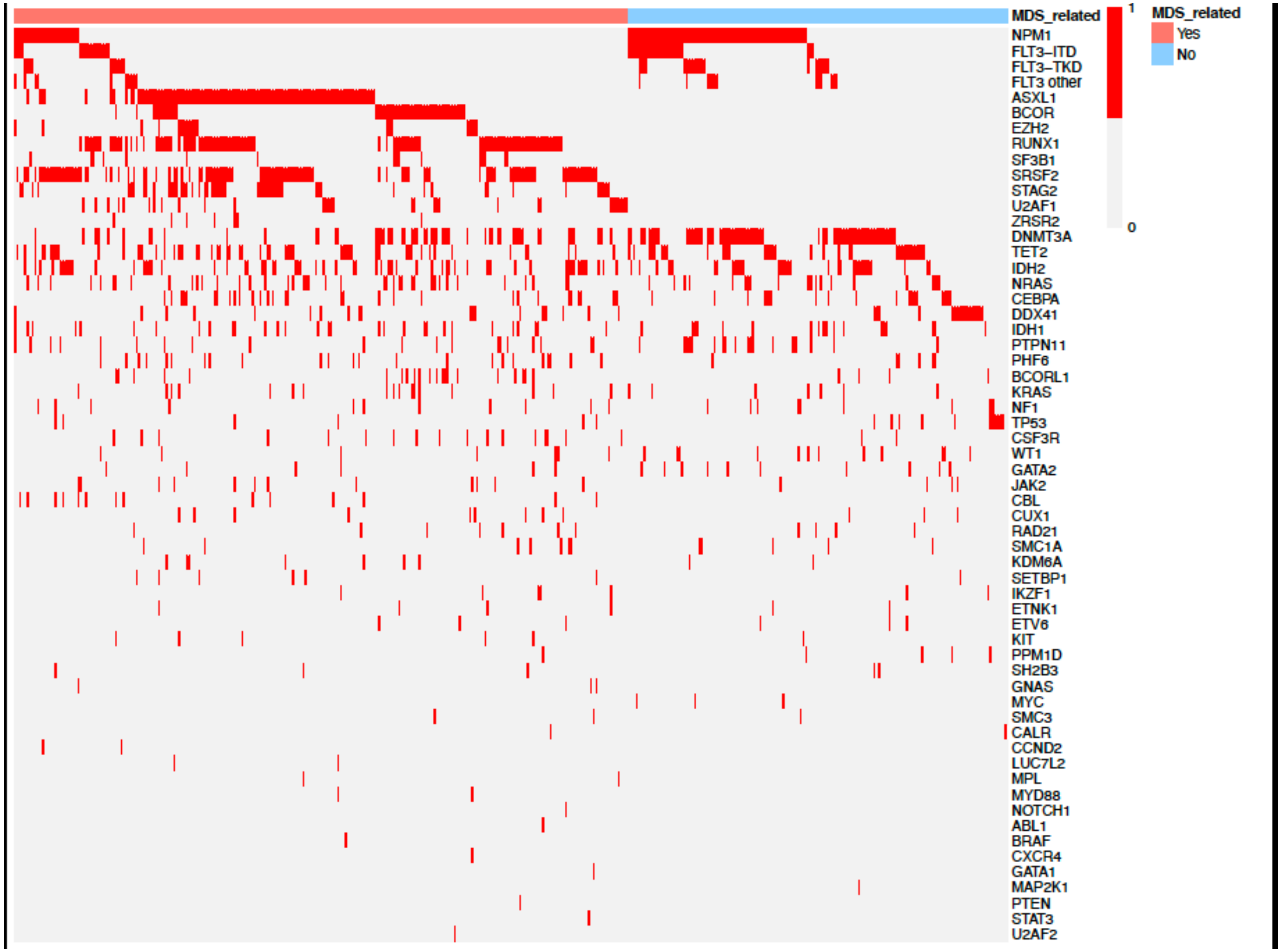

Supplementary Figure S2 A. Flow cytometric MRD levels in BM post course 1 according to treatment arm

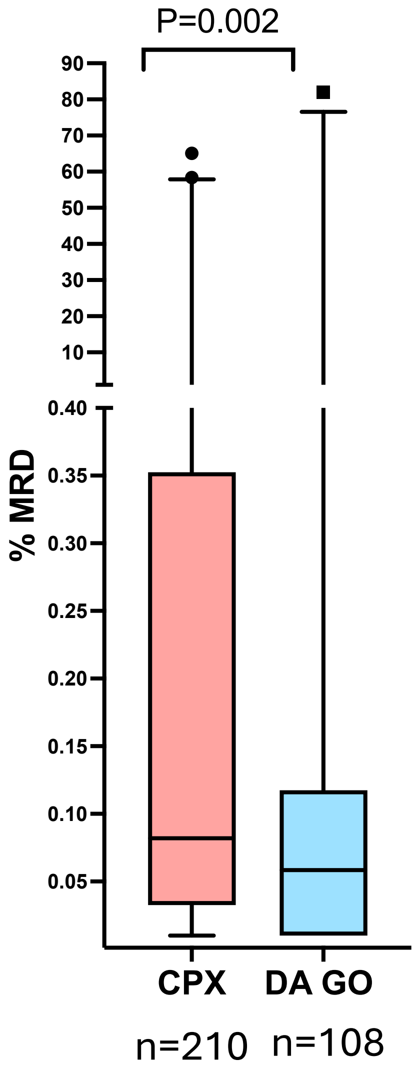

Supplementary Figure S2 B. Disposition of MRD data

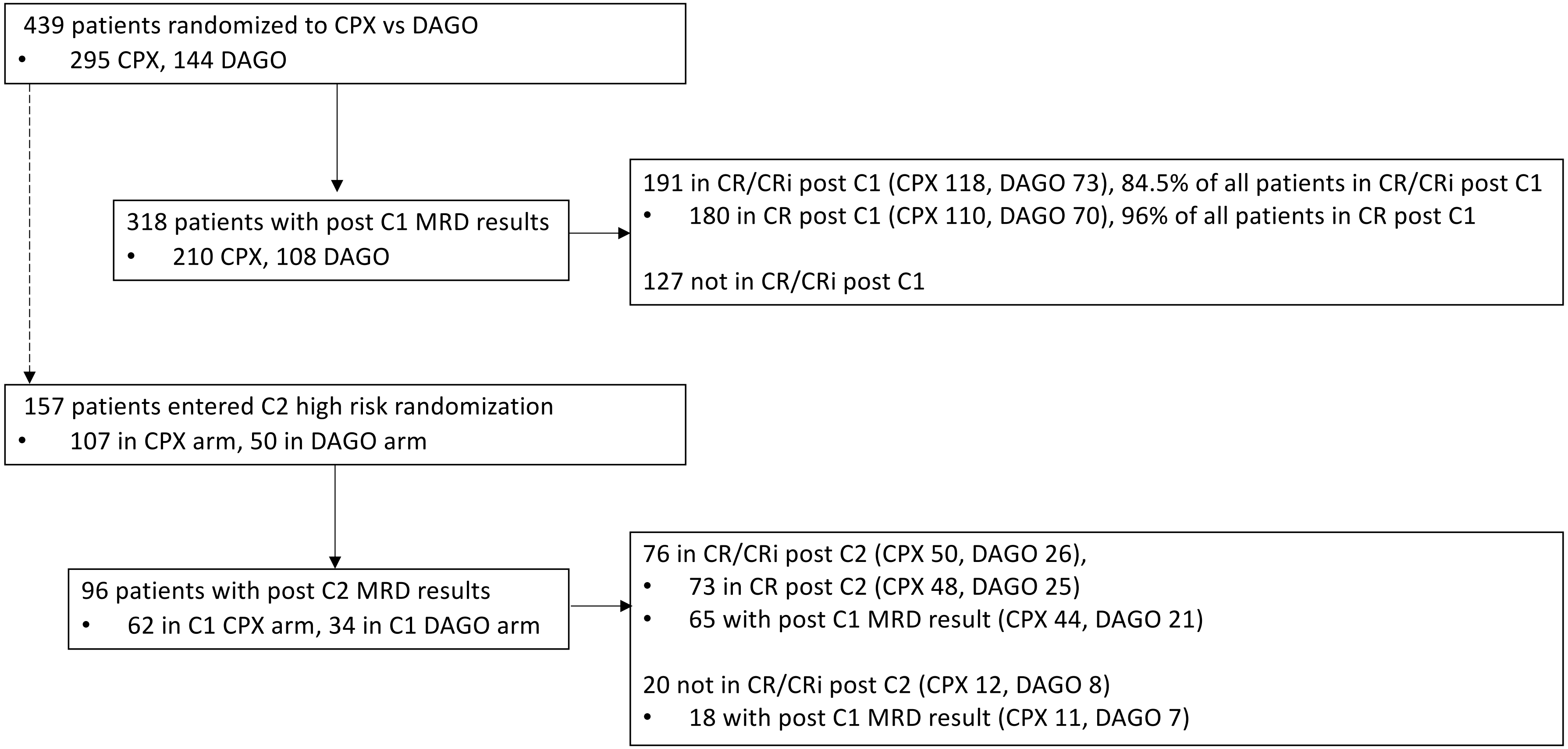

Supplementary Figure S2 C Flow Cytometric MRD response rates post course 1 by Molecular Subtypes

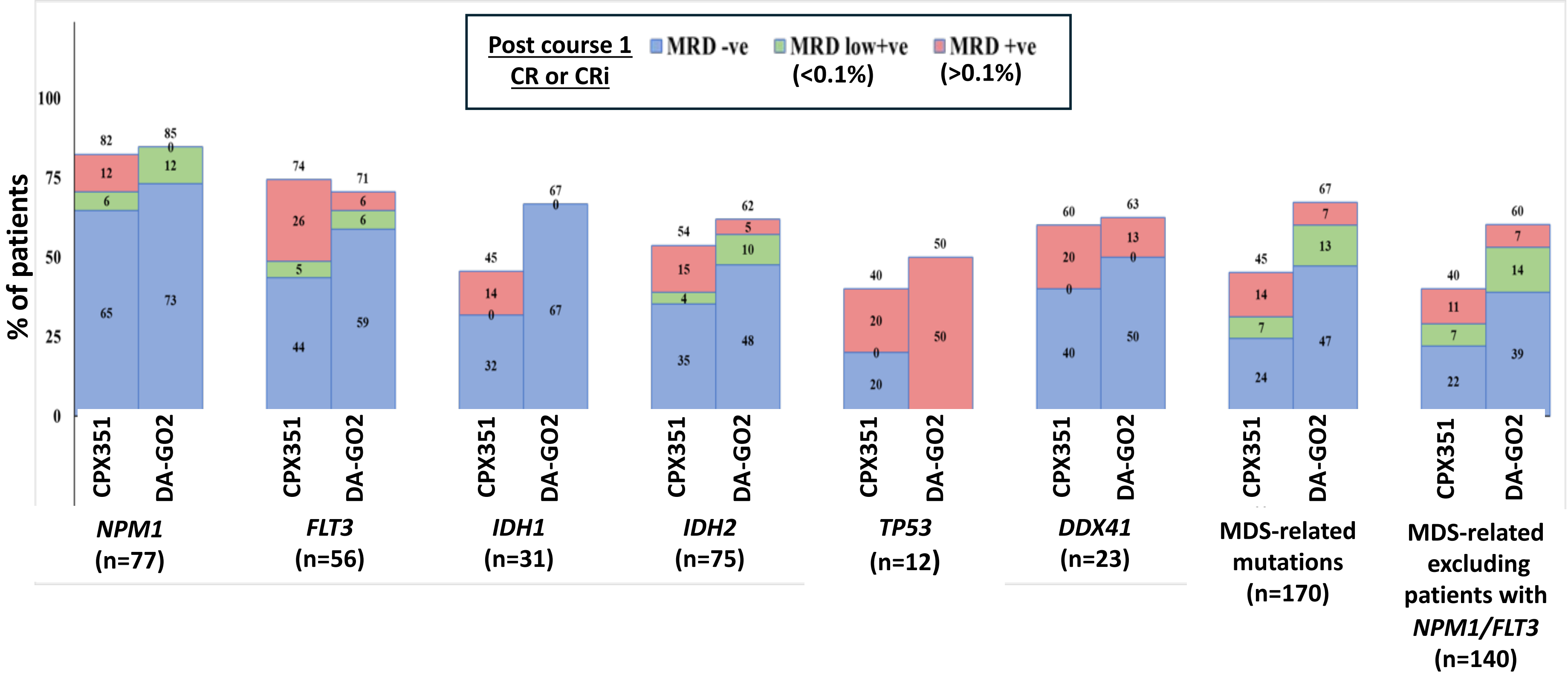

Supplementary Figure S3 Outcomes in patients achieving remission by day 50

A. Overall Survival

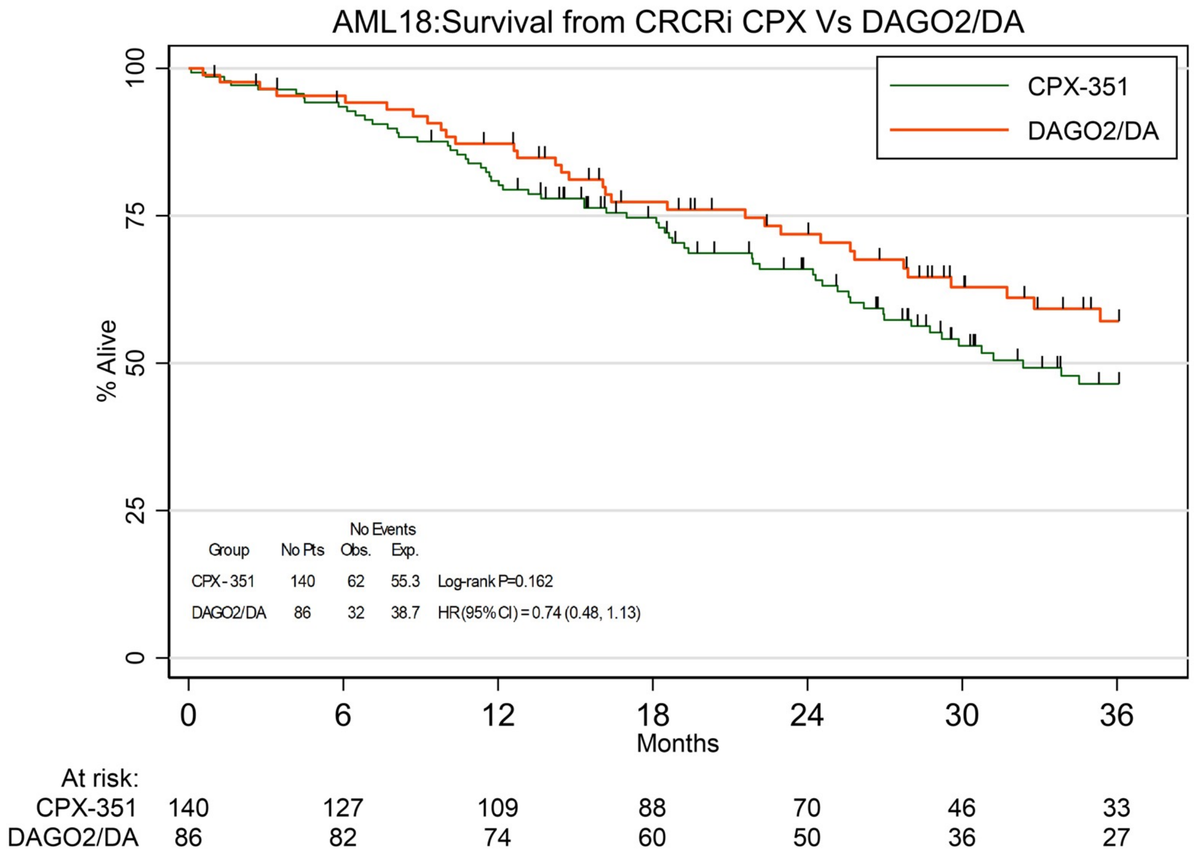

Supplementary Figure S3 Outcomes in patients achieving remission by day 50

B. Cumulative Incidence of Relapse

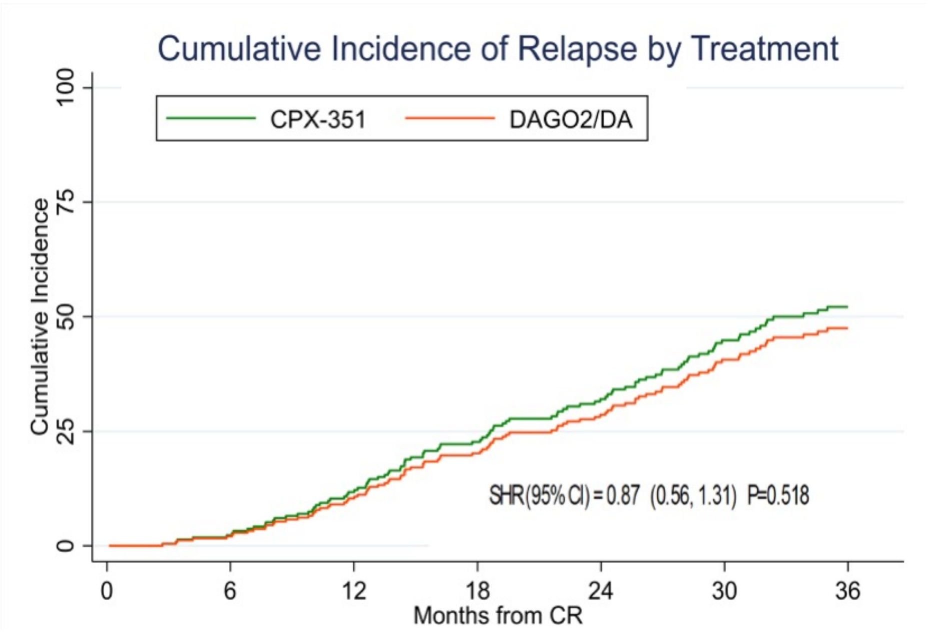

C. Cumulative Incidence of Death in Remission

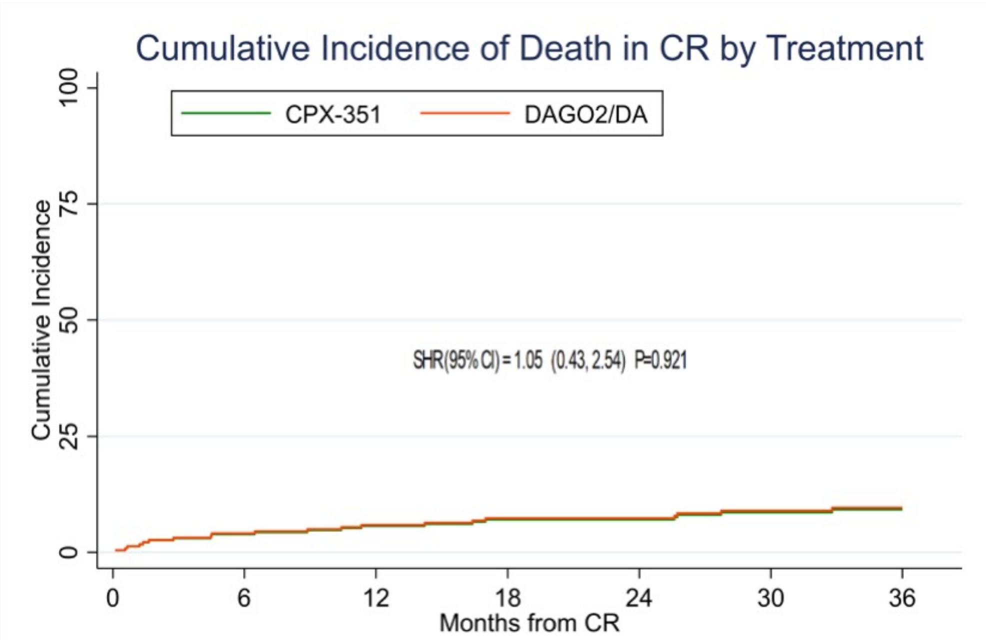

Supplementary Figure S4 Patients without adverse cytogenetics /TP53

A. Event Free Survival

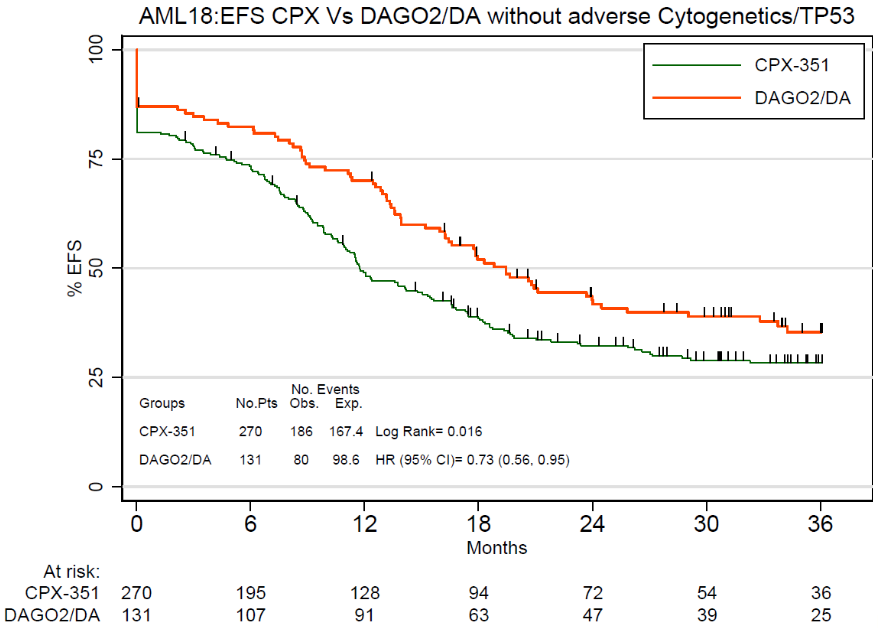

B. Overall Survival

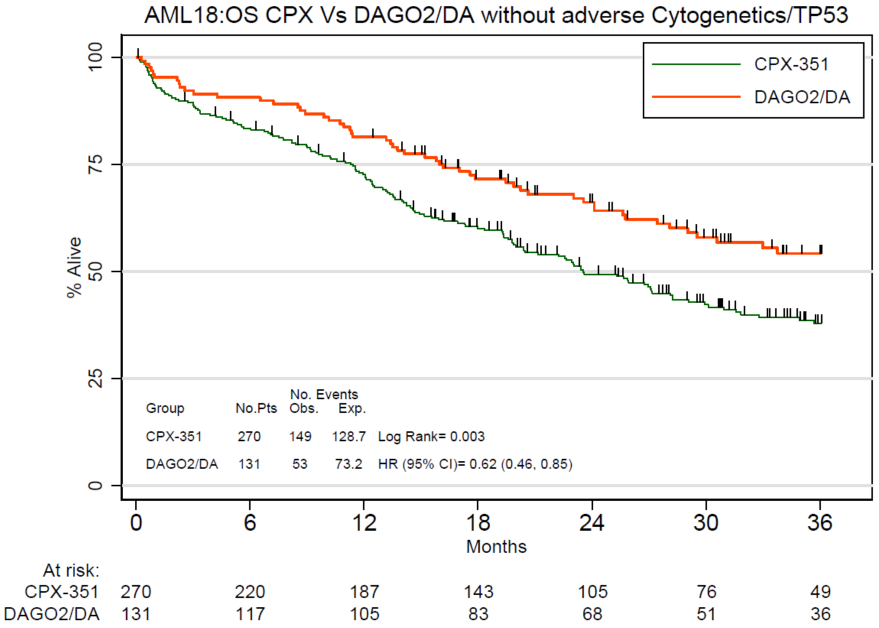

Supplementary Figure S5 Event free survival Subgroups

A.

AML18: Subgroup analysis EFS

Subgroup	DAGO2/DA	CPX-351	(O-E)	Var.	HR (95% CI) (CPX:DAGO2/DA)
**Age Group**					
<65 Yrs	21/43	55/86	-7.37	17.08	1.56 (0.94, 2.59)
65 - 69 Yrs	28/51	79/112	-13.23	23.43	1.78 (1.15, 2.75)
≥ 70 Yrs	40/50	74/97	0.77	24.21	0.97 (0.66, 1.43)
Test for Trend: Chi Sq=2.90 P value=0.089					
**Disease Type**					
De novo AML	71/117	171/240	-19.57	53.57	1.44 (1.09, 1.90)
Secondary AML	8/10	16/20	-0.38	4.60	1.07 (0.46, 2.51)
High Risk MDS	10/17	21/35	-1.02	6.88	1.16 (0.54, 2.46)
Test for Heterogeneity: Chi Sq=0.49 P value=0.484					
**ECOG ID**					
0	39/68	95/143	-8.13	29.40	1.32 (0.91, 1.92)
1	45/66	97/132	-9.44	31.00	1.34 (0.94, 1.91)
2	5/10	16/20	-3.89	4.67	2.40 (0.87, 6.62)
Test for Trend: Chi Sq=0.48 P value=0.490					
**SEX**					
Male	52/88	116/174	-8.74	36.83	1.26 (0.91, 1.75)
Female	37/56	92/121	-12.62	28.40	1.56 (1.06, 2.29)
Test for Heterogeneity Chi Sq=0.68 P value=0.411					
**WBC Group**					
<10	63/99	137/205	-7.08	43.55	1.17 (0.87, 1.58)
10 - 49.9	20/35	54/69	-11.62	16.26	2.05 (1.22, 3.44)
50+	6/10	17/21	-2.52	4.90	1.68 (0.66, 4.27)
Test for Trend: Chi Sq=0.32 P value=0.571					

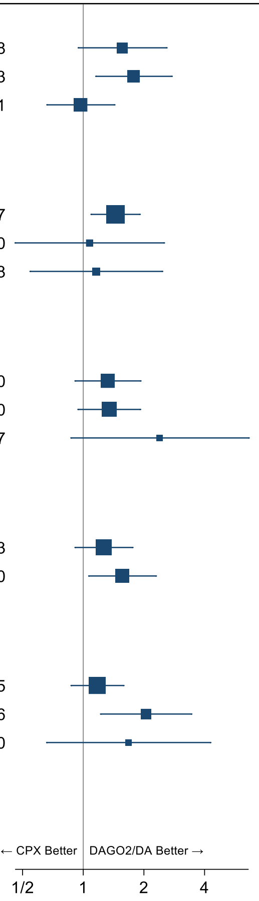

B.

AML18: Subgroup analysis EFS

Subgroup	DAGO2/DA	CPX-351	(O-E)	Var.	HR (95% CI) (CPX:DAGO2/DA)
**Cytogenetic**					
Favorable	2/2	2/2	0.33	0.72	0.62 (0.05, 7.00)
Intermediate	63/104	165/242	-16.11	49.19	1.39 (1.04, 1.86)
Adverse	8/12	18/20	-2.88	4.67	1.63 (0.70, 3.76)
**ELN 2022**					
Favorable	20/34	41/63	-5.40	14.27	1.47 (0.86, 2.51)
Intermediate	12/23	35/48	-6.25	10.20	1.87 (0.97, 3.62)
Adverse	40/56	95/133	-0.80	25.99	1.03 (0.71, 1.49)
**Molecular Marker**					
FLT3 all	12/21	37/45	-6.32	10.13	1.86 (0.96, 3.58)
NPM1 all	15/31	43/66	-7.97	13.29	1.89 (1.05, 3.42)
MDS Related Mutations	54/78	118/165	-4.91	36.04	1.14 (0.82, 1.57)

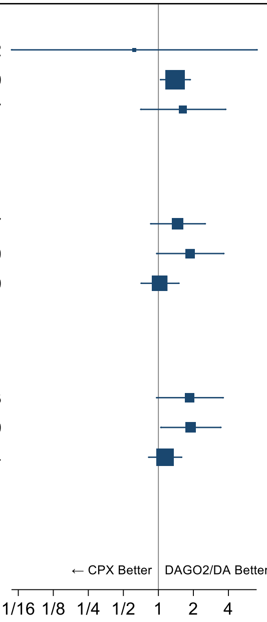

Supplementary Figure S6. Genetic Subgroup Outcome Analysis excluding patients receiving quizartinib

A. OS

AML18: Subgroup analysis OS

Subgroup	DAGO2/DA	CPX-351	(O-E)	Var.	HR (95% CI) (CPX:DAGO2/DA)
**Cytogenetics**					
Favorable	2/2	1/2	0.33	0.72	0.62 (0.05, 7.00)
Intermediate	42/104	138/242	-20.07	40.47	1.73 (1.23, 2.45)
Adverse	7/12	17/20	-2.63	5.68	1.64 (0.68, 3.98)
**ELN 2022**					
Favorable	14/32	34/63	-4.45	11.31	1.52 (0.81, 2.83)
Intermediate	7/20	30/48	-5.14	8.09	2.10 (0.92, 4.80)
Adverse	29/55	83/133	-5.30	23.75	1.26 (0.83, 1.93)
**Molecular Marker**					
FLT3 all	7/15	30/45	-2.57	7.06	1.50 (0.66, 3.42)
NPM1 all	6/26	29/66	-5.19	7.58	2.28 (0.95, 5.50)
MDS Related Mutations	39/76	103/165	-8.16	31.44	1.31 (0.91, 1.90)

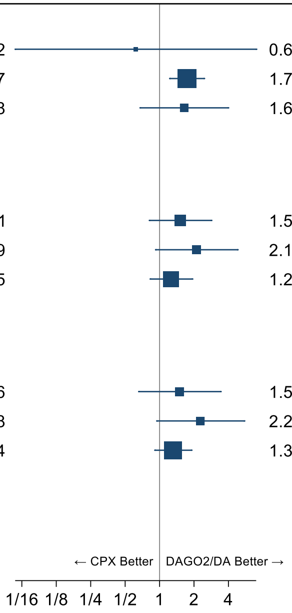

B. EFS

AML18: Subgroup analysis EFS

Subgroup	DAGO2/DA	CPX-351	(O-E)	Var.	HR (95% CI) (CPX:DAGO2/DA)
**Cytogenetic**					
Favorable	2/2	2/2	0.33	0.72	0.62 (0.05, 7.00)
Intermediate	63/104	165/242	-16.11	49.19	1.39 (1.04, 1.86)
Adverse	8/12	18/20	-2.88	4.67	1.63 (0.70, 3.76)
**ELN 2022**					
Favorable	20/32	41/63	-4.19	14.07	1.35 (0.79, 2.31)
Intermediate	12/20	35/48	-4.18	9.72	1.54 (0.80, 2.98)
Adverse	39/55	95/133	-1.33	25.82	1.05 (0.72, 1.52)
**Molecular Marker**					
FLT3 all	11/15	37/45	-1.93	8.41	1.24 (0.63, 2.44)
NPM1 all	15/26	43/66	-4.90	12.56	1.50 (0.83, 2.71)
MDS Related Mutations	54/76	118/165	-3.20	35.48	1.09 (0.79, 1.50)

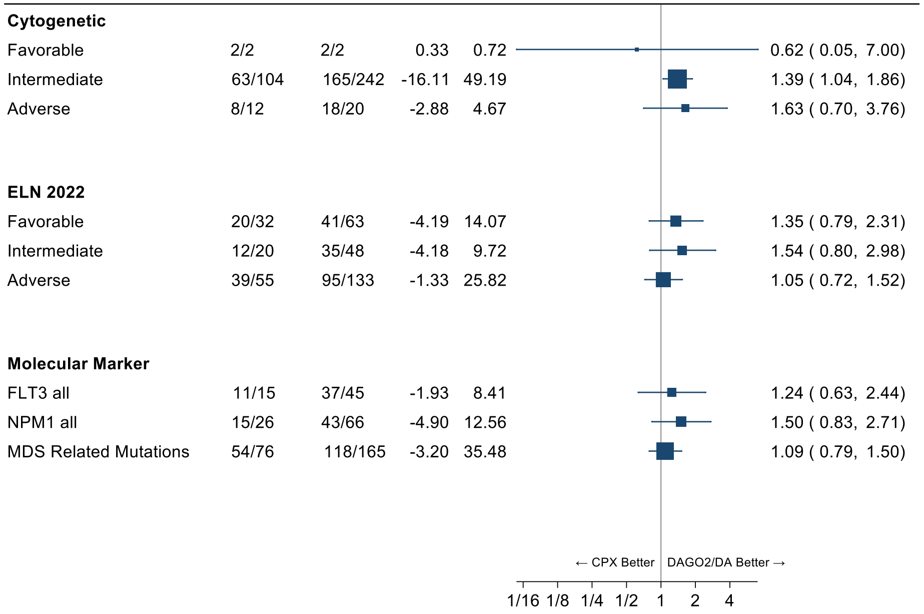

Supplementary Figure S7. Post transplant outcomes by Randomisation

A. Overall Survival

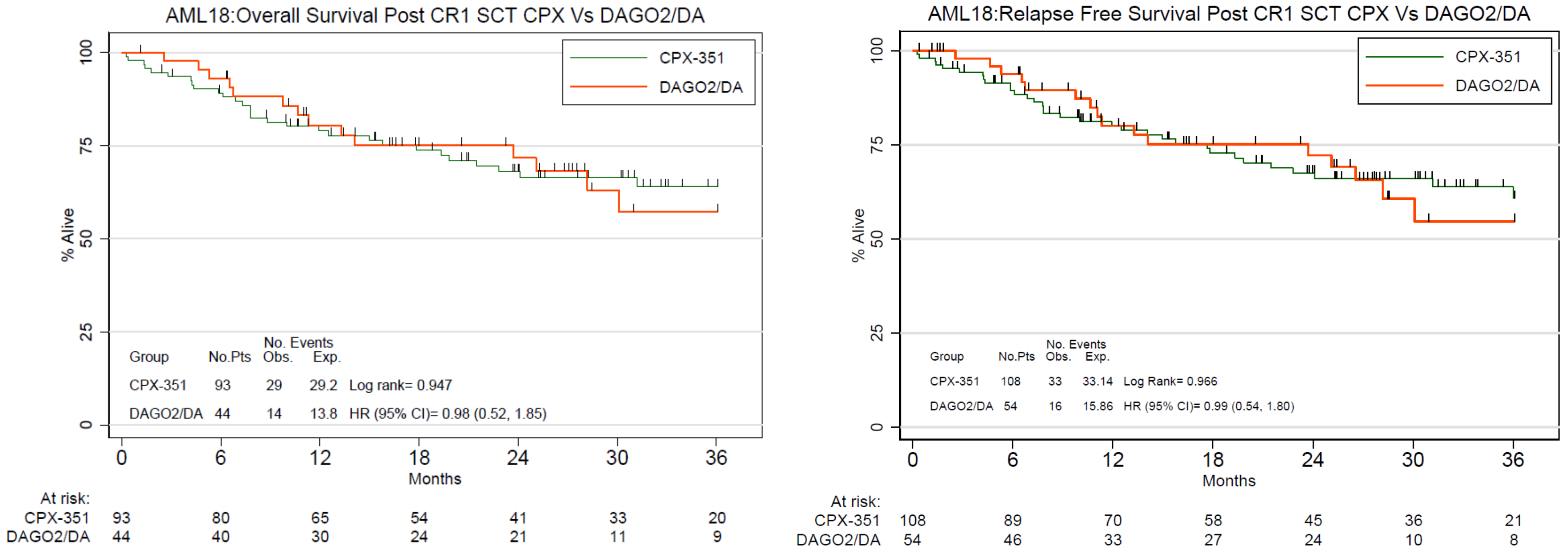

B. Relapse Free Survival

Supplementary Figure S8. Adverse events

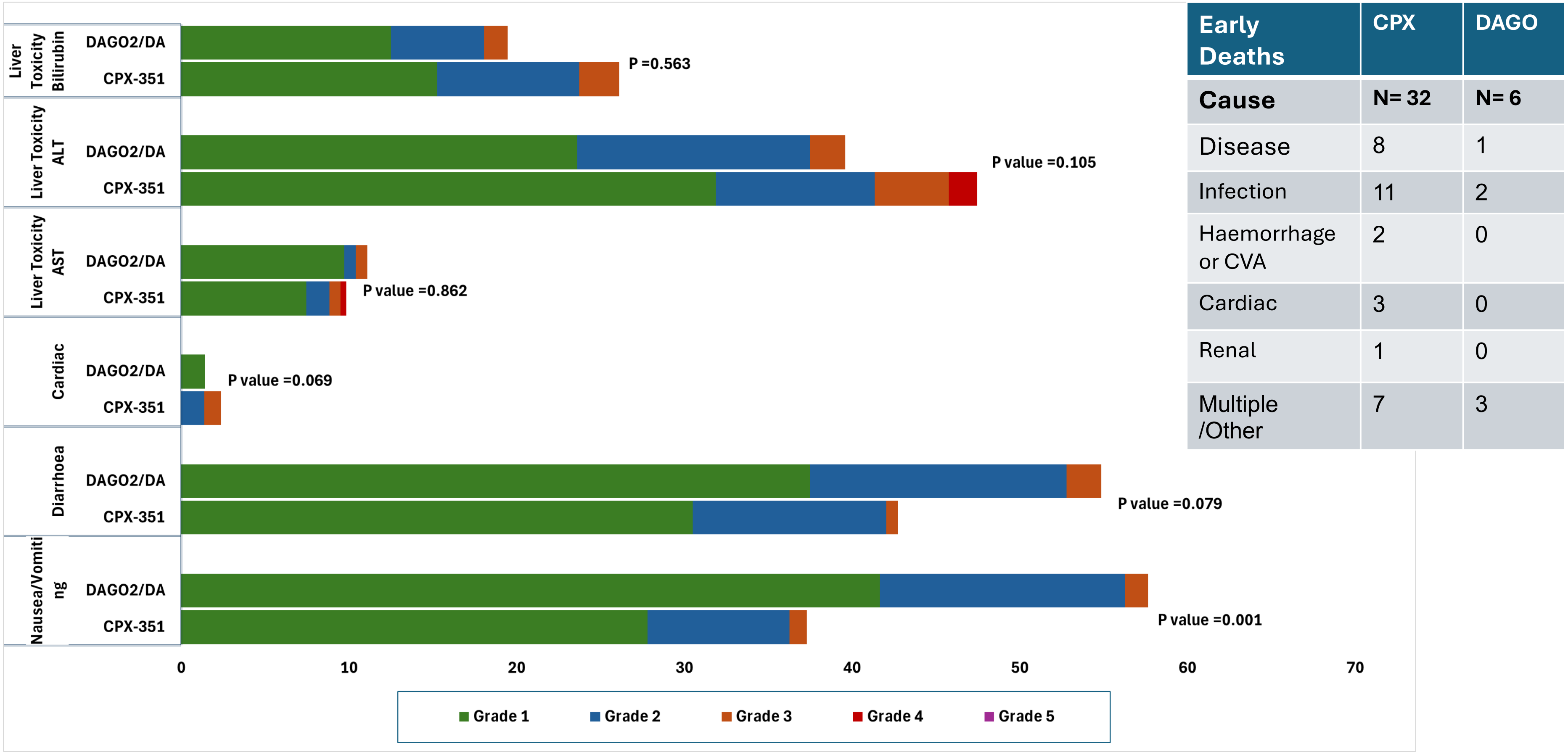

Early Deaths	CPX	DAGO
**Cause**	**N= 32**	**N= 6**
Disease	8	1
Infection	11	2
Haemorrhage or CVA	2	0
Cardiac	3	0
Renal	1	0
Multiple /Other	7	3

Supplementary Figure S9 Overall survival by CPX high risk course 2 randomization

A. All patients

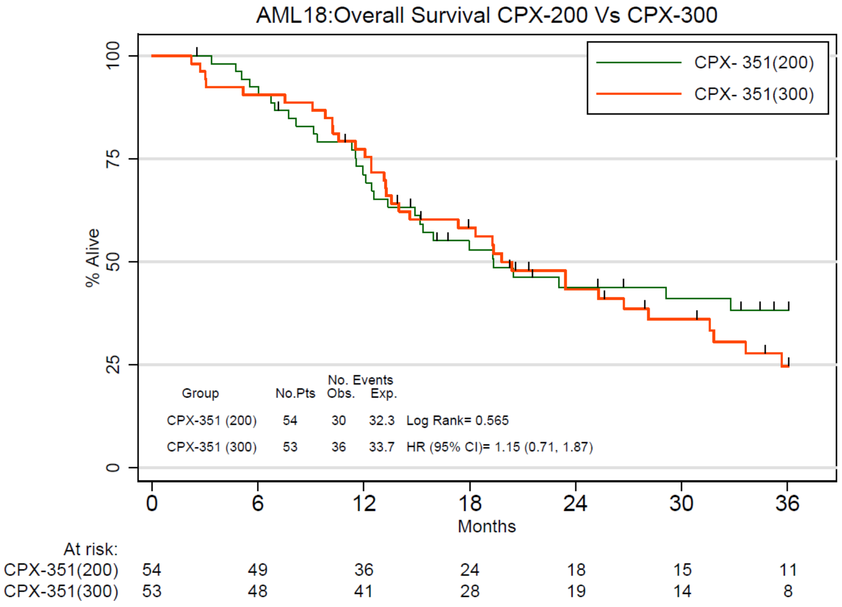

B. Excluding patients with unknown MRD status

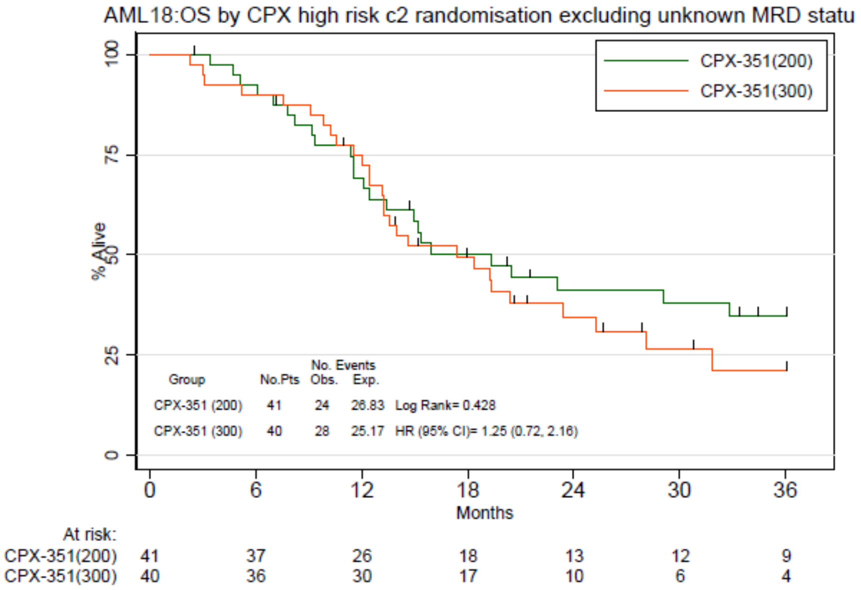

Supplementary Figure S10. Patient accrual (per month)

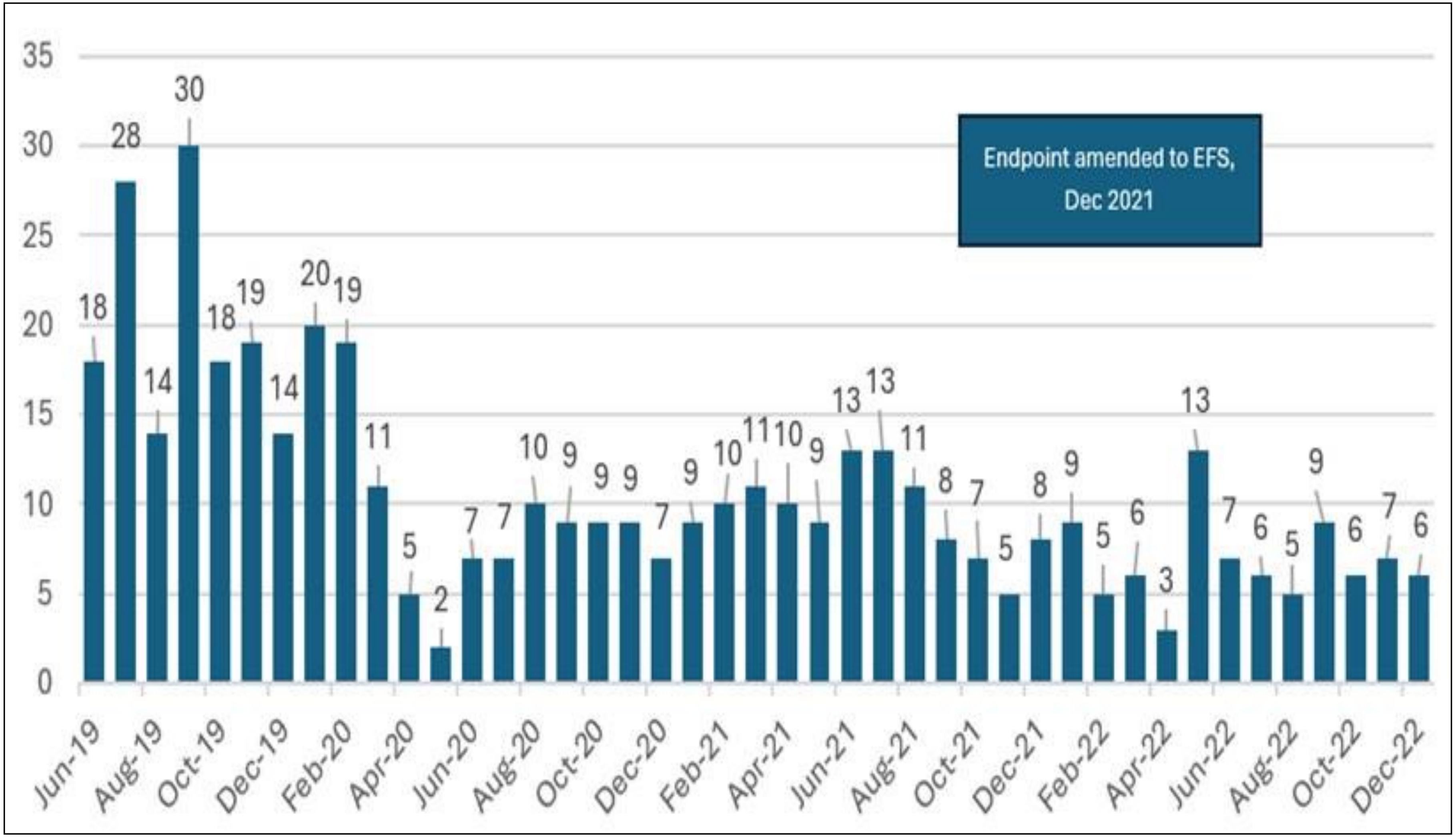

Supplementary Methods

DNA sequencing

Genomic DNA was extracted from archival blood or bone marrow samples stored at -80°C using the chemagic DNA Blood 2k kit H24 (cat# CMG-1097, Revvity) on the chemagic360 instrument (Revvity). DNA concentration was measured using the Quant-iT PicoGreen dsDNA Assay kit (cat# P11496, Thermo Fisher Scientific) on the Synergy LX microplate reader (BioTek Instruments, Inc.). DNA sequencing was performed using an error-corrected anchored multiplex PCR-based panel targeted 75 genes recurrently mutated in myeloid malignancies (Archer VariantPlex Myeloid Panel, Integrated DNA Technologies) as shown below.

ABL1	*DCK*	*KIT*	*RPS14*
ANKRD26	*DDX41*	*KMT2A*	*RUNX1*
ASXL1	*DHX15*	*KRAS*	*SETBP1*
ATRX	*DNMT3A*	*LUC7L2*	*SF3B1*
BCOR	*ETNK1*	*MAP2K1*	*SH2B3*
BCORL1	*ETV6*	*MPL*	*SLC29A1*
BRAF	*EZH2*	*MYC*	*SMC1A*
BTK	*FBXW7*	*MYD88*	*SMC3*
CALR	*FLT3*	*NF1*	*SRSF2*
CBL	*GATA1*	*NOTCH1*	*STAG2*
CBLB	*GATA2*	*NPM1*	*STAT3*
CBLC	*GNAS*	*NRAS*	*TET2*
CCND2	*HRAS*	*PDGFRA*	*TP53*
CDC25C	*IDH1*	*PHF6*	*U2AF1*

CDKN2A	*IDH2*	*PPM1D*	*U2AF2*
CEBPA	*IKZF1*	*PTEN*	*WT1*
CSF3R	*JAK2*	*PTPN11*	*XPO1*
CUX1	*JAK3*	*RAD21*	*ZRSR2*
CXCR4	*KDM6A*	*RBBP6*	

Libraries were prepared according to manufacturer's instructions, with modifications, utilizing pre- and post-PCR separation on liquid handling robots. 50-ng of gDNA was brought to 50-µL using 10mM Tris-HCl pH 8.0 and was subjected to DNA fragmentation, end repair, A-tailing, purification using SPRIselect reagent (cat# B23318, Beckman Coulter, Inc., and ligation with a universal ArcherDx molecular barcode (MBC) adapter, which tags each DNA molecule with a unique molecular index (UMI) and allows for unidirectional amplification of the sample using gene-specific primers. Following molecular barcode ligation, the libraries were subjected to two rounds of nested PCR for target enrichment. DNA fragmentation, end repair, cleanup after end repair, ligation step 1, cleanup after ligation step 1, MBC adapter incorporation, ligation step 2, cleanup after ligation step 2, and setup of first PCR were all performed on a Sciclone G3 NGS Workstation (Revvity). Cleanup after first PCR, second PCR, and cleanup after second PCR were all performed on a Zephyr G3 NGS Workstation (Revvity). Incubations and PCR reactions were performed on a Mastercycler X50a (Eppendorf). For the first PCR, amplification was performed as follows: 95°C for 3 minutes; 12 cycles of 95°C for 30 seconds, 62°C for 15 minutes; 72°C for 3 minutes. For the second PCR, amplification was performed as follows: 95°C for 3 minutes; 16 cycles of 95°C for 30 seconds, 65°C for 15 minutes; 72°C for 3 minutes.

The resulting libraries were subjected to paired-end 150-bp sequencing on a NovaSeq 6000 (Illumina), according to manufacturer's instructions. Libraries were pooled for sequencing such that each sample had a unique dual index. FASTQ files are available in the NCBI Short Reads Archive (SRA) (Accession: PRJNA1237261).

Raw sequencing FASTQ files were analyzed using the Archer Analysis software version 6.2.7 or 7.2.1, using default settings, except for the deep shallow threshold set to 3 (defining a deep error-correctable bin as ≥3 reads per molecular barcode/UMI). A median error-corrected depth of 2191X was achieved across samples.

DNA structural variation and SNP-InDel pipelines were utilized. The following variant filters were applied:

Variants in all genes (except *NPM1* and *FLT3*-ITD as described below) called by the *de novo* (LoFreq and Freebayes) and targeted (Vision) variant algorithms underwent a first round of filtering to: 1) remove anomalies generated as the result of library preparation and sequencing; and 2) select for variants associated with leukemic progression. The following filter conditions were applied in step 1:

1. Deep Alternate Observations (DAO) ≥5

2. Unique Start for Alternate Observation (UAO) ≥3

3. gnomAD AF ≤0.001

4. VEP Consequence = coding_sequence_variant, feature_elongation, feature_truncation, frameshift_variant, incomplete_terminal_codon_variant, inframe_deletion, inframe_insertion, missense_variant, protein_altering_variant, start_lost, stop_gained, stop_lost, transcript_ablation, transcript_amplification, splice_acceptor_variant, or splice_donor_variant, splice_region_variant

5. Has Sample Strand Bias = No

6. Has Seq Dir Bias = No

7. HRUN ≤9

8. ND Deep Allele Frequency (DAF) outlier P value ≤0.0001

9. Median distance from start site > 20

10. VAF ≥0.01

NPM1

Variants within the *NPM1* gene identified by the targeted (Vision) variant algorithm had the following filters applied:

1. Alternate Observations (AO) ≥3

2. VEP Consequence = frameshift_variant

3. VAF ≥0.01

***FLT3*-ITD**

Variants resulting in internal tandem duplications (ITD) within exons 14-15 of the *FLT3* gene were identified by either the structural variation algorithm (as described above) or by the de novo variant algorithm. For the structural variation algorithm, a minimum of 5 breakpoint-spanning reads were required to support the structural variation. For short ITDs (<20bp) identified by the *de novo* variant algorithm the following filters were applied:

1. Alternate Observations (AO) ≥3

2. VEP Consequence = inframe_insertion

3. VAF ≥0.01

All remaining variants underwent manual curation to select pathogenic or likely pathogenic variants and carried forward for further analysis (Table S4).

AML18 list of sites and investigators

The following investigators recruited patients:

Aalborg Hospital, Marianne T Severinsen, Anne Roug, Gitte Madsen, **Aarhus University Hospital**, Hans Beier Ommen, **Aberdeen Royal Infirmary**, Dr D J Culligan, Dr J Tighe, Dr Gavin Preston, Dr M Lamaechia, Manmeet Randhawa, Dr A. Lawrie, Thura Win Htut, Wail Abdelrahman, **Addenbrooke's Hospital**, Dr Faisal Basheer, Charles Crawley, Ben Uttenthal, Michael Chapman, Dr Kiran Tawana, **Aintree University Hospital**, Dr Vikram Singh, Dr Lynny Yung, Dr Jeffery Smith, Dr Vikram Singh, Dr Indrani Karpha, Dr Katherine Lindsay, **Arrowe Park Hospital (Wirral)**, Dr Ranjit Dasgupta, Dr Elizabeth Jones, Dr Barbara Hammer, Dr Giridharan Durgam, **Basingstoke and North Hampshire Hospital**, Dr Henna Wong, Harberth Fernandez-Leyva, Henna Wong, Nigel Sargant, Benjamin Gray, Ashok Roy, Kanchana De Abrew, Holly Gilbert, Hussain Janan, **Beatson General Hospital (Gartnavel General Hospital)**, Professor Mhairi Copland, Pam McKay, Edward Fitzsimons, Mark Drummond, David Irvine, Anne Parker, Richard Soutar, Mike Leach, Nicholas Heaney, Grant McQuaker, **Belfast City Hospital**, Dr C Arnold, Dr R Cuthbert, Dr C Arnold, Dr D Finnegan, Dr Bethany Mitchell, Stephen Boyd Dr N.Cunningham, Rachel Brockbank, **Birmingham Heartlands Hospital**, Dr Vidyha Murthy, Shankara Paneesha, Richard Lovell, Bhuvan Kishore, Charalampos Kartsios, **Blackpool Victoria Hospital**, Dr Paul Cahalin, Dr Seye Kolade, Dr. Raisa Guerrero, Dr Bosko Andjelic, Johnathan Elliot, Naeem Desai, Ahmed Abdulgawad, Thomas Seddon, **Bradford Royal Infirmary**, Dr Suresh Krishnan, Dr Anshu Garg, Dr Adrian Williams, Nandini Sadasivam, Abida Naeem, **Bristol Haematology & Oncology Centre**, Dr Priyanka Mehta, Dr Rachel Protheroe, Dr Sanne Lugthart, **Castle Hill Hospital**, Dr Simone Green, Dr Andrew Fletcher, Dr Senthilkumar Durairaj, **Cheltenham General Hospital**, Dr A Rye, Dr Richard Lush, Dr Asha Johny, Dr Rebecca Frewin, Dr Michael Shields, Dr Adam Bond, Dr R McCulloch, **Christie Hospital**, Dr Mike Dennis, Dr Mohd Mamat, Dr Dan Wiseman, **Churchill Hospital**, Prof Paresh Vyas, Andy Peniket, **Clatterbridge Cancer Centre (Royal Liverpool)**, Dr Arpad Toth, Dr Floisand, Countess Of Chester Hospital Dr Arvind Pillai, Dr Salaheddin Tueger, Dr Gillian Brearton, **Crosshouse Hospital (Ayrshire and Arran Health Board)**, Dr W Gordon, Peter Maclean, Mark MacColl, Paul Micallef Eynaud, Ian Devanny, Lea Haskins, Mark Rafferty, **Derby Teaching Hospital**, Dr I Amott, Dr A Glover, Adrian Smith, Juanah Addada, Tom Taylor, Firas Al-Kaisi, Malik Saeed, **Derriford Hospital (Plymouth)**, Dr Patrick Medd, Simon Rule, Claire Hutchinson, Wayne Thomas, Tim Nokes, David Lewis, Sophie Johns, Rory McCulloch, Loredana Mihailescu, Sarah Mant, Gerorgina Symes, **Forth Valley Hospital**, Dr Hugh Edwards, Dr Rachel Boulton-Jones, Dr Katrina Farrell, Dr Sarah Barcroft, Dr. Hanlon, Dr R F Neilson, **Freeman Hospital (Newcastle)**, Dr Gail Jones, Dr Nicola Redding, Prof Graham Jackson, Dr Tobias Menne, Dr Wendy Osborne, Dr Erin Hurst, Dr Andrew Charlton, Dr Helen Marr, Dr Michelle Lannon, Dr Andrew McGregor, Dr Tom Fail, **Gloucestershire Royal Hospital**, Dr Adam Rye, Dr Richard Lush, Dr Asha Johny, Dr Rebecca Frewin, Dr Mike Shields, Dr Philip Robson, Dr Sally Chown, **Guy's Hospital**, Dr Richard Dillon, **Herlev University Hospital**, Claudia Schöllkopf, Mette Borg Clausen, Daniel El Fassi, Syed Azhar Ahmad, Bo Kok Mortensen, Nina Toft, **Hillingdon Hospital**, Dr Richard Kaczmarski, Dr Taku Sugai, Dr Akila Danga, Dr Ketan Patel, Dr Katherine Bailey, **Ipswich Hospital**, Dr Mahesh Prahladan, Dr Debo Ademokun, Dr Andrew Hodson, Dr Isobel Chalmers, Dr Ioana Whalley, **James Cook University Hospital**, Dr R Dang, Dr Diane Plews, Dr Jamie Maddox, Dr Angela Wood, Dr Marianna David, Dr Mohsen Norouzi, **Kettering General Hospital**, Dr Mark Kwan, Dr Jag Gandla, Dr Isaac Wilson-Morkeh, Dr AURANGZEB RAZZAK, Dr Rebecca Allchin, Dr Alex Gebreyes, Dr Matthew Player, **Leeds Teaching Hospital NHS Trust (St James)**, Dr Richard Kelly, Dr Anjum Khan, Dr Manish Jain, Prof David Bowen, Melvish Ul-Haq, **Leicester Royal Infirmary**, Dr Katherine Hodgson, Dr Murray Martin, Anna Tsoulkani, **Lincoln County Hospital**, Dr Charlotte Kallmeyer, Dr Osama Ali, Dr Gamal Sidra, Dr Ali Boden, Lauren Henry Dr Pavel Chudakou Dr Rajendra Pol, **Maidstone Royal Infirmary**, Dr Evangelia Dimitriadou, Dr Lalita Banerjee, Dr Richard Gale, Dr Clare Wykes, Adrian Maraj, **Manchester Royal Infirmary**, Dr Eleni Tholouli, Dr Fiona Dignan, Dr Sarah Burns, Caroline Wall, Margarita Triantafillou, Thomas Seddon, Srividhya Senthil, Ahmed Amer, **Milton Keynes University Hospital**, Dr Moez Dungarwalla, Dr Subir Mitra, Dr Sarah Davis, Dr Magbor Akanni, **Musgrove Park Hospital (Taunton & Somerset Hospital)**, Dr Deepak Mannari, Simon Bolam, Belinda Austen, Lisa Lowry, **New Cross Hospital**, Dr Richard Whitmill, Dr

Suprahk Basu, Dr Richard Whitmill, Dr Sophie Lee, **New Victoria Hospital**, Dr Gail Loudon, Dr Ian MacDonald, Dr Alistair Hart, **NHS Lanarkshire (Monklands, Wishaw and Hairmyres Hospital)**, Dr Christopher McDermott, Dr Linsay Mitchell, Dr Pamela Paterson, Dr Iain Singer, Dr Adrew Fyfe, Dr Charlotte Thomas, Dr Jane Laird, Dr Alice Cooke, Dr Annielle Hung, **Ninewells Hospital (Dundee)**, Dr Sudhir Tauro, Gordon Marron, Duncan Gowans, Raed-Alkhatib, Michelle Harrison, **Norfolk and Norwich University Hospital**, Dr Angela Collins, Dr Matthew Lawes, Prof Kris Bowles, Dr Nimish Shah, Dr Charlotte Hellmich, Dr Cesar Gomez, Dr Joel Cunningham, Victoria Willimott, **Northampton General Hospital**, Dr Jane Parker, Dr Sajjan Mittal, Dr Alistair McGrann, **Nottingham University Hospital**, Dr Jenny Byrne, Gerardo Errico, Jyoti Nangalia, Ruth Witherall, Prof Nigel H Russell, **Pinderfields General Hospital**, Dr Joanna Haughton, Dr David Wright, Dr John Ashcroft, Dr Kavita Patil, Dr Joanna Haughton, Dr Clare Kane, Dr William Wong, Dr Paul Moreton, Dr Muhammad Mohsin, **Poole General Hospital**, Dr Darshayani Furby, Dr Ram Jayaprakash, Dr Rebecca Maddams, Dr Louise Fraser, Dr Alistair Smith, Dr Bhoomika Roomalah, **Queen Alexandra Hospital (Portsmouth)**, Dr R Corser, Mary Ganczakowski, Robert Corser, Robert Ayto, Charle Alderman, Kanchana DeAbrew, Dr Gwynn Matthias, Emily Robinson, Dr Edward Belsham, **Queen Elizabeth Hospital Birmingham**, Vidhya Murphy, Dr Manos Raghavan, Malahat Saeed, Richard Buka, Dr Himabindu Rebbapragada, Dr Justin Loke, Dr Clare Lodwick, Dr Phillip Nicolson, **Queen's Hospital, Romford**, Dr Paul Greaves, **Raigmore Hospital**, Dr Caroline Duncan, Dr Jo Craig, Dr Peter Forsyth, Dr Katherine Leighton, Dr Frances Buckley, **Rigshospitalet University Hospital**, Ulrik Malthe Overgaard, Ove Juul Nielsen, Kim Theilgaard-moench, Kirsten Grønbæk, Peter Kampmann, Eva Funding, Eva Leinøe, Dr Lars Kjeldsen, **Roskilde Hospital**, Peter Møller, Morten Krogh Jensen, **Royal Bournemouth General Hospital**, Dr David Allotey, Renata Walewska, Helen McCarthy, Kunaal Kaushik, Gavinda Sangha, Rachel Hall, David Allotey, Dr Ifraz Hamid, Marcin Lubowiecki, **Royal Cornwall Hospital (Treliske)**, Dr Bryson Pottinger, Dr David Tucker, Ruth Witherall, Elizabeth Parkins, Adam Forbes, **Royal Devon & Exeter Hospital**, Dr Thomas Coates, Dr Jackie Ruell, Dr Paul Kerr, Dr Loretta Ngu, Dr Anthony Todd, **Royal Free Hospital**, Dr P Kottaridis, Dr Kate Cwynarsky, Dr Chris McNamara, Dr Mallika Sekhar, Dr Ashu Wechalekar, **Royal Marsden Hospital**, Dr David Taussig, Dr Mark Ethell, Dr Mike Potter, Dr Chloe Antheas, Nma-Okike Nzekwu, Dr Emma Nicholson, Dr Thinzar Ko Ko, Dr Patrick Elder, **Royal Oldham Hospital**, Dr David Osborne, Dr Allameddine Allameddine, Dr Hayley Greenfield, Dr Martin Rowlands, Dr Antonina Zhelyazkova, Dr Choudhuri Satarupa, Dr Atanas Stanchev, Dr Muhammad Pervaiz, **Royal Stoke University Hospital**, Dr Srivinas Pillai, Dr Richard Chasty, Dr Neil Phillips, Dr Kamaraj Karunanithi, **Royal United Hospital Bath**, Dr Chris Knechtli, Dr Sarah Wexler, Dr Josephine Crowe, Dr Sally Moore, Dr Gihan Mahmoud, Dr Rhys Williams, Dr Joanna Collins, **Russells Hall Hospital**, Dr Rupert Hipkins, Dr Stephen Jenkins, Dr Craig Taylor, Dr Jeff Neilson, Dr Avio Fernandes, Dr Ovine Gamage, Dr Yadanar Lwin, **Salford Royal Hospital**, Dr Rowena Thomas-Dewing, Dr Simon Jowitt, Dr Clare Barnes, Dr Mark Henry, **Salisbury District Hospital**, Dr Jonathan O Cullis, Dr Effie Grand, Dr Sally Bugg, Dr Tracey Parker, Dr James Milnthrope, **Sandwell Hospital**, Dr Farooq Wandroo, Dr Yasmin Hasan, Dr Richard Murrin, Dr Shivan Pancham, Dr John Gillson, Dr Wright, Dr Pancham, Dr Hisam Siddiqi, Dr S Altaf, **Singleton Hospital (Swansea)**, Dr Unmesh Mohite, Dr Hamdi Sati, Dr Hamid Majid, Dr Rhian Jones, **Southampton General Hospital**, Dr D Richardson, Dr Kim Orchard, Dr Matthew Jenner, Dr Srinivasan Narayanan, Dr Christopher Dalley, Dr Thomas Cummin, Dr Josh Dmochowski, **St Bartholomew's Hospital**, Dr Heather Oakervee, Dr Jamie Cavenagh, **St Helier Hospital**, Dr Simon Stern, Dr Caroline Ebdon, Dr Roslin Zuha, Dr Sneha Muthalali, Dr Stella Appiah-Cubi, Dr Corinne De Lord, Dr Emily Bart-Smith, **St Richard's Hospital**, Dr Santosh Narat, Dr Jamie Wilson, Dr Heba Yassin, Dr Salah Alhassan, **Sunderland Royal Hospital**, Dr Scott Marshall, Dr Victoria Hervey, Dr Yogesh Upadhye, Dr Annete Nicolle, Dr Susanna Mathew, Dr Emily Graves, Dr Alexander Langridge, Dr Shikha Chattree, **Torbay District General Hospital**, Dr Rui Zhao, Dr Loredana Mihailescu, Dr Heather Eve, Dr Patrick Roberts, Dr Rui Zhao, Dr Barry Jackson, **University College London Hospital**, Dr Rob Sellar, Dr Vicky Stables, Wei Yee Chan, William Townsend, Suzanne Arulogun, Catriona Mactier, Nisha de Silva, **University Hospital Odense**, Duruta Weber, Claus Werenberg Marcher, Andreja Dimitrijevic, Klas Raaschou-Jensen, Maria Kallenbach, Mette Levring, **University Hospitals Coventry and Warwickshire**, Dr Beth Harrison, Dr Benjamin Bailiff, Dr Louise Fryearson, Dr Jhansi Muddana, Dr E Tebbet, Dr R. Afghan, Dr K. Randall, Dr Dunca Murray, Dr Anton Borg, Dr Francesca Jones, Dr Sarah Nicolle, Dr Salama Abosaad, Dr Maria Mushkbar, **University Hospital of Wales (Cardiff)**, Dr S Knapper, Dr Jonathan Kell, Dr Caroline Alvares, **Western General Hospital (Edinburgh)**, Dr Vicotria Campbell, Dr Huw Roddie, **Wexham Park Hospital**, Dr Mark Offer, Dr Simon Moule, Dr

Nicola Bienz, Dr Nicola Philpott, Dr Carolina Lahoz, **Whiston & St Helens Hospital**, Dr Toby Nicholson, Dr J Tappin, Dr David Taylor, Dr Sammy Fergiani, Eleana Loizou, Ushma Meswani, Sally Evans, **Worcestershire Acute Hospitals NHS Trust**, Dr Nicholas Pemberton, Dr Clare Rowan, Dr Juliet Mills, Dr Safia Jalal, Dr Thomas Skibbe, Dr Iman Quereshi, Dr Khin Cho Thein, Dr Tracey Chan, **Worthing Hospital**, Dr. Santosh Narat, Andrew Wood, Dr Ronwyn Cartwright, Dr Santosh Narat, **York Hospital**, Dr Muhammad Naveed, Dr Lee Bond, Dr Jennifer Shields, Dr Laura Munro, Dr Annika Whittle, **Ysbyty Glan Clwyd**, Dr Earnest Heartin, Dr Yvonne Jones, Dr Margaret Goodrick, Dr Steph Jackson, Dr Durgadevi Moratuwagama, **Ysbyty Gwynedd**, Dr Ernest Heartin, Dr Chris Williams, Dr David Edwards
